# Optimized topology control for large-scale IoT networks using graph-based localization

**DOI:** 10.1038/s41598-026-43621-6

**Published:** 2026-03-17

**Authors:** Indrakshi Dey, Nicola Marchetti

**Affiliations:** 1 Walton Institute, South East Technological University, Waterford, Ireland; 2 Trinity College Dublin, Dublin, Ireland

**Keywords:** Internet of things (IoT), Node localization, Graph realization, Topology design and control, Eigenvector synchronization, Engineering, Mathematics and computing

## Abstract

Internet of Things (IoT) is increasingly realized through large scale deployments of heterogeneous devices and gateways operating under strict energy budgets and interference limited links, which motivates reliability aware topology control and end to end communication performance objectives. As IoT deployments grow to massive scales and incorporate highly heterogeneous devices, designing and controlling network topology in a reliable and energy-efficient manner becomes a fundamental challenge. In particular, poor link quality, interference, and localization uncertainty severely limit the effectiveness of traditional topology-control approaches. In this paper, we address this challenge by introducing IoTNTop, a novel and unified graph-based framework for joint localization, graph embedding, and topology control in large-scale, resource-constrained IoT networks. Unlike conventional methods that decouple localization from topology design, IoTNTop embeds both end-nodes and gateways into a globally consistent spatial structure using partial and noisy distance measurements, and directly couples this geometry with communication-aware topology optimization. IoTNTop adopts an error-centric topology-control objective that explicitly minimizes end-to-end (E2E) error probability while enforcing practical code-rate and transmit-power constraints. The framework jointly optimizes link activation, transmit power, and data transmission code rate, and employs a scalable sub-graph stitching pipeline based on eigenvector synchronization (EVS), landmark alignment (LA), and semidefinite programming (SDP) refinement. A greedy signal-to-noise-ratio (SNR)–guided edge selection strategy with convergence checking further ensures computational efficiency. Comprehensive numerical analysis and network-level simulations show IoTNTop retains approximately 60–80% of the initial per-node energy budget while maintaining symbol error probability below 15% for the majority of nodes. At the same time, it converges in fewer iterations than Genetic Algorithm (GA) and brute-force baselines and sustains higher achievable code rates at lower transmit power levels. These performance gains remain consistent across the tested signal-to-noise ratio regimes and network sizes.

## Introduction

The rapidly expanding Internet of Things (IoT), featuring billions of heterogeneous devices across smart cities industrial automation and remote healthcare, faces unprecedented challenges in managing vast dynamic and resource constrained networks. As deployments grow in size and diversity, IoT networks must operate under strict energy limits while meeting application requirements on reliability data fidelity and timeliness. These demands make topology formation and control a central design issue, since link activation transmit power and coding rate choices directly shape interference decoding feasibility and long term network operation^1,2^.

Early research in wireless sensor networks (WSNs) established important foundations for distributed connectivity and energy aware operation. However the assumptions that often made WSN era solutions effective are increasingly misaligned with modern IoT deployments. Contemporary IoT systems are typically larger, more heterogeneous and more dynamic with a mix of constrained end nodes and more capable gateways operating in interference limited environments. In such settings, measurement noise link variability and incomplete observability are not secondary effects but primary constraints. As a result approaches that treat the network as homogeneous or that rely on stable well observed links can produce topologies that appear adequate under simplified models, yet fail to deliver reliable communication under practical conditions^3,4^.

This shift also changes the relationship between localization and topology control. In large scale IoT many decisions depend on inferred geometry, because distance driven path loss affects signal to noise ratio and therefore achievable code rate and error probability. When position estimates are inaccurate the resulting propagation estimates become biased, which can lead to selecting links that are nominally feasible but unreliable in practice. At the same time topology and power control decisions influence which links remain observable and which measurements remain informative over time. This creates a tight coupling where localization uncertainty and topology decisions interact rather than forming a clean pipeline. Consequently decoupled designs that first localize and then optimize topology often fail to propagate uncertainty into link selection power assignment and code rate choice. The outcome can be networks that preserve connectivity but do not meet end to end reliability targets under energy and coding constraints^3,4^.

A further limitation is that much prior work optimizes proxy objectives such as connectivity node degree or energy alone instead of directly targeting an error centric objective that matches communication performance. In modern IoT with low power wide area networks and mission critical workloads, the priority is often low error probability and reliable decoding under tight energy budgets and interference constraints^3,4^. In addition existing embedding pipelines can struggle to jointly place gateways and end nodes with global consistency when distances are partial and noisy, leaving a gap between spatial inference and link level performance at deployment scale^5^.

These gaps motivate a unified framework that couples geometric inference with reliability aware topology control. In this paper we introduce *IoTNTop*, a graph based framework that jointly localizes nodes and configures network topology by determining active links transmit power and code rate, so as to minimize end to end error probability under practical power and coding constraints^1,2^. Unlike conventional pipelines that separate localization from topology design IoTNTop embeds both end nodes and gateways into a globally consistent spatial structure, using partial and noisy distance measurements and directly incorporates this geometry into topology optimization (in this paper, “embed/embedding” refers to graph embedding for localization: constructing a globally consistent geometric realization (node positions for gateways and end-nodes) from partial and noisy pairwise distance information and overlaps between local sub-graphs. It does not refer to graph representation learning that compresses nodes or graphs into abstract feature vectors for downstream predictive tasks. Our use of *embedding* is strictly geometric and serves to recover the spatial layout that underpins path loss, SNR, and code-rate feasibility in the subsequent topology-control step). Specifically, IoTNTop (i) constructs a scalable geometric embedding from partial and noisy distance measurements using eigenvector synchronization (EVS), landmark alignment (LA), and semidefinite programming (SDP) refinement, and (ii) performs topology control by jointly selecting links, transmit power levels, and code rates to minimize end-to-end (E2E) error probability under realistic constraints. An SNR-guided greedy strategy with convergence checking is employed to align early iterations with decoding feasibility while maintaining computational efficiency. The main contribution of this work is a joint optimization framework that integrates localization, graph embedding, and topology control in a principled, error-centric manner. In particular, this paper makes the following contributions:

- Unified graph-based modeling for joint localization and topology control: we present a single framework that integrates geometric inference of heterogeneous IoT end-nodes and gateways with topology optimization under realistic communication constraints.
- Error-centric topology optimization: we formulate a topology-control objective that minimizes E2E error probability while enforcing code-rate feasibility and transmit-power limits, explicitly linking decoding reliability to link activation.
- Scalable multi-stage embedding under partial and noisy measurements: we develop a noise-resilient embedding pipeline that decomposes the network into sub-graphs, stitches them via EVS and LA, and refines the global geometry using SDP to ensure consistency.
- IoTNTop algorithm with convergence-aware optimization: we propose an iterative algorithm that employs SNR-guided link selection and convergence checks to efficiently steer the topology toward reliable configurations.
- Practical relevance for large-scale IoT deployments: the proposed framework is designed to accommodate device heterogeneity, limited energy budgets, and evolving network conditions without relying on exhaustive search or data-intensive learning.

Unlike our earlier work MaxNTop^6^, which focuses on maximizing node and link activation, IoTNTop is driven by an error-centric, code-rate-constrained objective coupled with power-aware link selection and globally consistent geometric embedding. The remainder of the paper is organized as follows. “System model” introduces the system model. “Related works and problem definition” reviews related work and formalizes the problem definition. “Network graph modelling” presents the proposed IoTNTop framework in detail. “Topology design and control” and “Numerical results and discussion” evaluate its performance through numerical analysis and simulation studies. “Simulation results and discussion” concludes the paper.

## System model

We consider a large-scale IoT network deployed over a two-dimensional geographic region and composed of heterogeneous wireless devices. The network includes two types of entities: *end-nodes* and *gateways*—interconnected through wireless links subject to power, noise, and reliability constraints. This section defines the entities, measurements, and physical-layer assumptions that underpin the subsequent problem formulation and algorithm design.

Network entities and geometry—let $\mathcal {V} = \mathcal {L} \cup \mathcal {S}$ denote the set of all nodes, where $\mathcal {L}=\{1,\dots ,l\}$ represents IoT end-nodes (e.g., sensors, meters, actuators) and $\mathcal {S}=\{l+1,\dots ,l+s\}$ represents gateways (anchors). Gateways are assumed to have higher energy and computational resources and may possess known or partially known locations, whereas end-nodes are resource-constrained and typically have unknown positions. Each node $i \in \mathcal {V}$ is associated with an unknown spatial coordinate ${\bf x}_i \in \mathbb {R}^2$ (or $\mathbb {R}^3$ where stated explicitly). The network geometry is inferred from partial and noisy measurements and serves as the basis for topology control and communication optimization.

Links and distance measurements—wireless connectivity is represented by an undirected graph $G=(\mathcal {V},\mathcal {E})$, where $(i,j) \in \mathcal {E}$ denotes a candidate wireless link between nodes *i* and *j*. Due to range and energy limitations, only a subset of all node pairs is observable, resulting in partial connectivity. For each feasible link $(i,j) \in \mathcal {E}$, a noisy distance measurement

1 $$\begin{aligned} d_{ij} = \Vert {\bf x}_i - {\bf x}_j\Vert + \varepsilon _{ij} \end{aligned}$$

is available, where $\varepsilon _{ij}$ captures measurement noise and environmental uncertainty arising from ranging, receiver signal strength (RSS)-based estimation, or other proximity-sensing mechanisms.

Channel, power, and reliability model—communication follows a distance-dependent path-loss model. Let $p_i$ denote the transmit power of node *i*, and let $G_{ij}$ represent the channel gain between nodes *i* and *j*. The received signal-to-noise ratio (SNR) on link (*i*, *j*) is given by:

2 $$\begin{aligned} \textrm{SNR}_{ij} = \frac{p_i G_{ij}}{N_0 + I_{ij}}, \end{aligned}$$

where $N_0$ denotes noise power and $I_{ij}$ denotes aggregate interference. Each node is subject to a transmit-power constraint $0 \le p_i \le p_i^{\max }$. Each active link supports an achievable code rate $R_{ij}$ determined by its SNR. A link is considered feasible if both $\textrm{SNR}_{ij}$ and $R_{ij}$ satisfy predefined reliability thresholds. The link reliability is strictly quantified by the *symbol error probability*, denoted as $P_e(i,j)$. We define $P_e(i,j)$ as the conditional probability that a transmitted symbol *s* is decoded incorrectly as $\hat{s} \ne s$ at the receiver, given the instantaneous channel quality. Formally, this is expressed as a strictly decreasing function of the SNR: $P_e(i,j) = \mathbb {P}(\hat{s} \ne s \mid \textrm{SNR}_{ij}) = \mathcal {F}(\textrm{SNR}_{ij})$. Here, $\mathcal {F}(\cdot )$ represents the generalized physical layer transfer function that maps the received $\textrm{SNR}_{ij}$ to an error probability. This function captures the fundamental performance characteristic of the link such that increasing transmit power $p_i$ (and thus $\textrm{SNR}_{ij}$) monotonically reduces $P_e(i,j)$, directly linking topology control decisions to decoding reliability.

## Related works and problem definition

### Background

Database-matching (DB-M) has become a viable localization method in IoT networks by comparing real-time signal measurements (e.g., Received Signal Strength Indicator (RSSI), Time-of-Arrival (ToA), or Angle-of-Arrival (AoA)) with a pre-established database of signal fingerprints from known locations^7^. Traditional DB-M techniques use probabilistic models or distance-based metrics to find the closest match^8,9^. However, DB-M requires extensive fingerprint databases that must be frequently updated—rendering them unsuitable for mobile or evolving IoT deployments. To enhance accuracy, machine learning (ML) methods have been applied. Key ML techniques include Artificial Neural Networks (ANN), which capture complex non-linear relationships^10,11^, Random Forests for robustness against noise and overfitting^12,13^, and Deep Reinforcement Learning (DRL), particularly useful in dynamic environments that require database updates^14,15^. However, ML approaches often demand high amounts of computational resources and large labeled datasets, which are typically unavailable or impractical for low-power, heterogeneous IoT nodes. These models also struggle to generalize to unseen environments or react to dynamic topology changes.

The authors in^6^ are the first to combine node localization with topology control for power optimization, using noisy distance measurements and eigenvector synchronization. The network topology is constructed via linear programming to maximize spatial usage and throughput, emphasizing accurate localization for power-efficient IoT design. Under matched conditions used in our experiments, $N=100$ nodes uniformly deployed over a $5\times 5\,\textrm{km}^{2}$ field and an average transmit power of $5\,\textrm{dB}$ per node (Figs. 9, 10, 11, 12), our proposed IoTNTop attains higher achievable code rates, lower symbol-error probabilities, and better per-node energy retention than heuristic and exhaustive baselines. In particular, most nodes maintain symbol error probability $<15\%$ (Fig. 15), and the network retains 60–$80\%$ of the initial per-node energy budget (Fig. 16) while sustaining higher code rates across iterations and network sizes (Fig. 12). These trends persist for $N\in [50,300]$ (Figs. 12, 13, 14, 15, 16). By contrast, MaxNTop^15^ optimizes node/edge activation but does not incorporate an error-centric, code-rate-constrained objective; *IoTNTop*’s joint optimization of power, code rate, and error probability yields superior operating points under the same deployment assumptions.

In^16^, localized algorithms allow nodes to make decisions based on neighborhood information, utilizing techniques like iterative power adjustment and node state switching for energy conservation. Decentralized solutions are highlighted for scalability in large networks. In^17^, Graph Signal Processing (GSP) is used to treat network measurements as graph signals, enabling spectral analysis and filtering to enhance localization and topology inference. However, GSP and other above-mentioned techniques, rely on assumptions of linearity and stationarity, which limits their applicability under high-noise or non-ideal conditions commonly observed in real-world wireless systems. On the topology control side, traditional strategies frequently emphasize energy savings or basic connectivity, while neglecting performance metrics like error probability, link reliability, or transmission rate–all of which are essential for modern IoT applications.

Hybrid approaches combine the strengths of different localization methods to overcome their individual limitations. Kalman Filtering and Bayesian techniques are often used to fuse data from various sources for probabilistic location estimates. Advanced approaches, such as game-theoretic^18^ and optimization-based models^23^, attempt to jointly manage transmit power and connectivity. However, these typically optimize for a single objective—such as network lifetime or throughput—while ignoring trade-offs involving link quality, error resilience, and coding efficiency. Most importantly, few existing methods integrate localization uncertainty into the topology design process, leaving a critical disconnect between spatial inference and performance optimization.

In addition to localization methods, a wide range of studies has explored topology control for IoT networks, with a focus on improving energy efficiency, maintaining connectivity, and enhancing fault tolerance. Classical approaches based on Minimum Spanning Tree (MST)^24^ and Local Minimum Spanning Tree (LMST)^25^ offer low-complexity link pruning but fail to incorporate dynamic environmental factors or communication noise. Clustering protocols like Low Energy Adaptive Clustering Hierarchy (LEACH)^26^ and Hybrid Energy-Efficient Distributed (HEED)^27^ introduce hierarchical structures to reduce interference and manage scalability, yet often assume homogeneous node capabilities and rely on periodic re-clustering, which limits adaptability in diverse network settings.

Existing approaches typically decouple localization from topology control, leaving measurement uncertainty unpropagated into link selection and routing; optimize energy or throughput in isolation rather than minimizing error probability under code-rate constraints; and rely on embeddings that do not jointly place gateways and end-nodes with guarantees of global consistency under partial and noisy distances. Moreover, quantitative analyses of convergence and computational complexity under realistic power and SNR budgets are limited. These gaps motivate an integrated, error-centric formulation that jointly optimizes power, code rate, and error probability and employs a scalable EVS and LA with SDP embedding tailored to large, noisy IoT deployments.

**Fig. 1:**
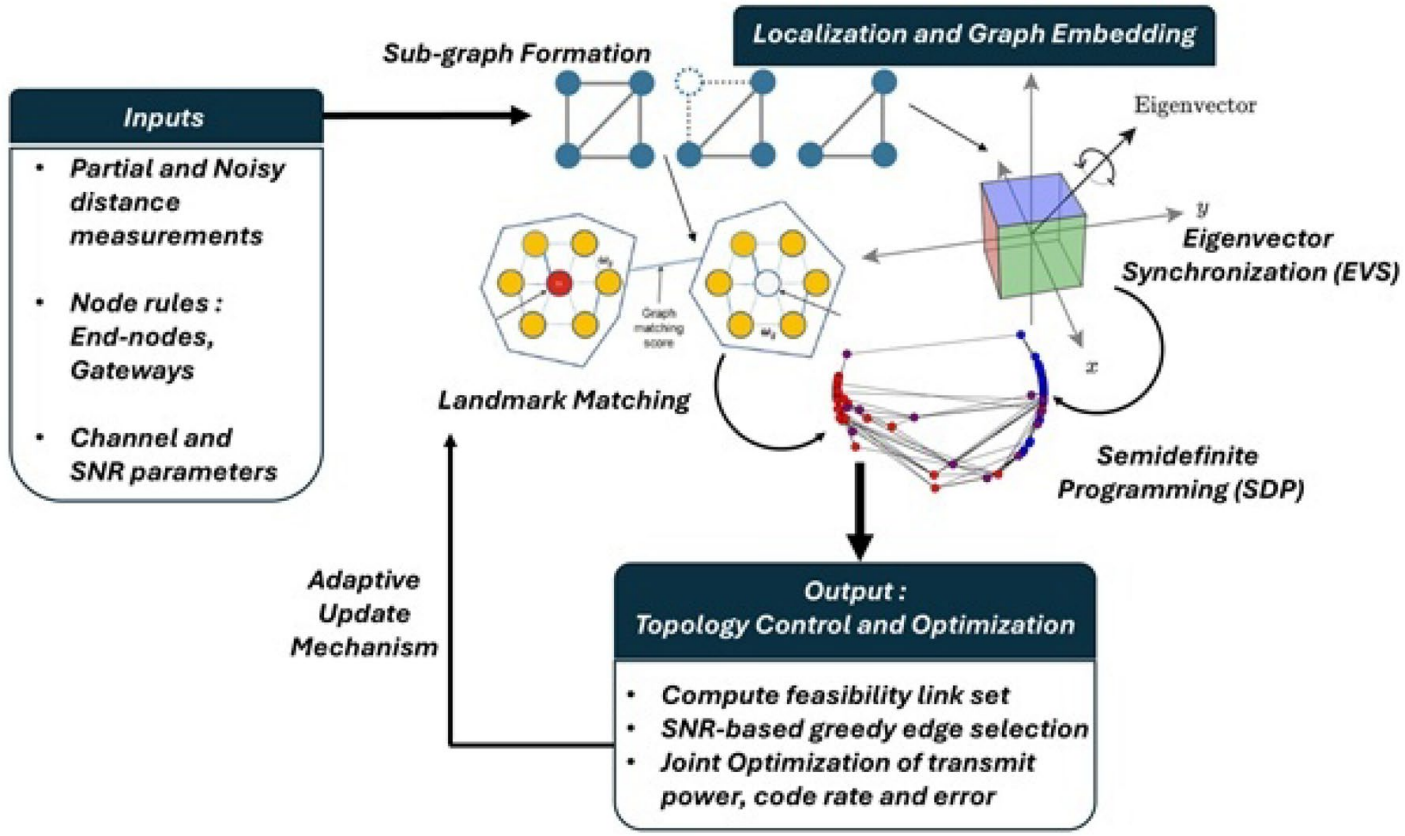
A conceptual overview of proposed optimized topology control. Different colors represent different types of IoT nodes.

### Problem definitions and scope

Building upon the system model described in “System model”, we now formalize the joint optimization problem addressed by *IoTNTop*. The overarching objective is to design a reliable and energy-efficient IoT network by tightly coupling spatial inference with topology control. This problem is conceptually decomposed into four interacting layers: *localization*, *graph embedding*, *topology control*, and *topology design*, which function sequentially to bridge the gap between noisy physical measurements and reliable communication.

Localization: the process begins with the challenge of estimating node positions from partial and noisy pairwise distance measurements, $\mathcal {D}=\{d_{ij}:(i,j)\in \mathcal {E}\}$, along with the coordinates of any available gateways. The primary goal at this stage is to derive local coordinate estimates, denoted as $\hat{{\bf x}}_i$, for nodes within overlapping network neighborhoods. Because these estimates are derived from incomplete data, they are not initially guaranteed to be globally consistent; local sub-graphs may differ from the true global geometry by rigid transformations such as reflection, rotation, and translation. Correcting these local ambiguities is critical, as localization errors directly distort propagation loss estimates, thereby compromising the accuracy of link feasibility, SNR calculations, and achievable code rates in subsequent control stages.

Graph embedding: to resolve the inconsistencies inherent in local estimates, the graph embedding stage reconciles the localized sub-graphs into a single, globally consistent geometric realization $\hat{{\bf x}}=\{\hat{{\bf x}}_i\}_{i\in \mathcal {V}}$ for both end-nodes and gateways. *IoTNTop* achieves this through a scalable, multi-stage pipeline designed to progressively align the network geometry. First, the pipeline employs *EVS* to resolve global reflection and rotation ambiguities. Conceptually, EVS is a spectral method that constructs a matrix representing the relative transformations between sub-graphs and computes its leading eigenvector to recover the consistent global orientation of each patch. Following orientation, the pipeline performs *LA* to correct rigid translation and scaling discrepancies. This technique estimates the optimal rigid transformations by minimizing the sum of squared Euclidean differences between shared nodes (landmarks) across overlapping sub-graphs. Finally, an *SDP* refinement step is applied to enforce global distance consistency across the stitched network. The result is a unified coordinate embedding that serves as the precise geometric substrate required for effective topology control.

Topology control: once the network geometry is embedded, the topology control layer determines the optimal configuration for network operation. Using the inferred global coordinates $\hat{{\bf x}}$, channel parameters, and device power limits, this stage jointly selects the active link set $\mathcal {E}^\star \subseteq \mathcal {E}$, assigns transmit powers $\{p_i\}$, and determines link code rates $\{R_{ij}\}$. *IoTNTop* distinguishes itself by adopting an *error-centric* objective, which explicitly minimizes the network-level end-to-end (E2E) error probability subject to strict decoding feasibility and power constraints. Central to this objective is the link-level symbol error probability, $P_e(i,j)$, which quantifies the reliability of a connection. We define $P_e(i,j)$ as the probability that a transmitted symbol is decoded incorrectly at the receiver, a value computed analytically from the instantaneous link SNR according to the modulation-dependent error model. By aggregating these link-level probabilities, the control algorithm steers the network toward configurations that maximize global reliability.

Topology design: while topology control optimizes parameters for the current epoch, *topology design* enforces longer-term structural preferences that must persist over time. This layer specifies the admissible solution classes through constraints such as minimum coverage requirements, bounded node degrees, gateway attachment rules, or sparsity targets. These regularization terms guide the optimizer toward feasible configurations that satisfy high-level architectural goals beyond immediate error minimization.

Interfaces and information flow: the four components interact sequentially and iteratively. Partial and noisy distance measurements first drive the localization and graph embedding layers to recover the global geometry. This reconstructed geometry dictates the physical channel properties, specifically pathloss and SNR, which define the decoding feasibility of potential links. These constraints then feed into the topology control layer, which optimizes power and code rates to minimize error. The resulting topology decisions update the link feasibility and measurement availability for the subsequent epoch. Figure 1 provides a system-level illustration of this information flow, highlighting the interdependence of spatial inference and reliability-aware control within the framework.

Coverage definition: to ensure network service quality, we define coverage based on signal quality rather than simple connectivity. A node *i* is considered covered if it possesses at least one neighbor *j* in the feasible link set $\mathcal {E}_f$ such that the signal quality meets a minimum standard, $\textrm{SNR}_{ij} \ge \textrm{SNR}_{\textrm{thr}}$. The network-wide coverage ratio is formulated as

$$C = \frac{1}{|\mathcal {V}|}\sum _{i\in \mathcal {V}} {\bf 1}\big (\exists j:(i,j)\in \mathcal {E}_f,\ \textrm{SNR}_{ij} \ge \textrm{SNR}_{\textrm{thr}}\big ).$$

A minimum threshold $C_{\min }$ is enforced as a hard constraint during optimization to guarantee sufficient network availability. The primary decision variables governing this process are the node coordinates ${\bf x}$, transmit powers ${\bf p}$, active link set $\mathcal {E}^\star$, and code rates ${\bf R}$. Collectively, these formulations define the joint optimization landscape where spatial inference and topology control converge, providing the necessary theoretical basis for the algorithmic solution presented in the following section.

## Network graph modelling

Under the system and problem definitions above, *IoTNTop* operates on a known set of nodes $\mathcal {V}$ with fixed roles (end-nodes and gateways). What is unknown are the node coordinates and the active communication topology. At each epoch, the algorithm is provided with partial, noisy distance measurements $\mathcal {D}=\{d_{ij}\}$ rather than a full distance matrix. The candidate link set is defined as

$$\mathcal {E}_f = \{(i,j): \textrm{SNR}_{ij}(p_i)\ge \textrm{SNR}_{\textrm{thr}},\ p_i\le p_i^{\max }\},$$

encoding links that are feasible under the power budget. The goal of *IoTNTop* is twofold: (i) to estimate a globally consistent geometric embedding from $\mathcal {D}$, and (ii) to design and control the active topology $\mathcal {E}^\star \subseteq \mathcal {E}_f$ jointly with transmit powers and code rates to minimize end-to-end error probability. To achieve scalability, the network graph is decomposed into overlapping sub-graphs. Sub-graphs sharing sufficient common nodes are aligned through reflection, rotation, and translation to remove geometric ambiguities before being stitched into a global embedding. This process enables reliable topology inference and control in large, noisy IoT deployments. Figure 1 illustrates the resulting workflow, in which heterogeneous clusters are progressively aligned and integrated into a unified global graph that reflects both spatial structure and communication feasibility.

### Graph-embedding for end-nodes

Let us first decompose the graph *G* into smaller, overlapping sub-graphs. Let these initial sub-graphs be denoted by $P_1, P_2, \ldots , P_N$, where *N* is the total number of sub-graphs created from the decomposition, and each $P_k \subseteq G$ represents a local patch or sub-network formed by a subset of nodes and their immediate edges. Let $p_i=[x_i,y_i]^T$ denote the spatial coordinate vector of node *i*; in particular, $p_n$ is the position of node *n*, and $p_1$ is the position of node 1. Let $P_k=[p_{i_1},p_{i_2},\dots ,p_{i_m}]$ denote the set of node position vectors belonging to subgraph *k*. Uppercase symbols ($P_k$) refer to subgraph-level embeddings (collections of node locations), whereas lowercase symbols ($p_i$) refer to individual node locations. Each $P_k$ is obtained by locally realizing the nodes of subgraph *k* from the available partial/noisy pairwise distances; the resulting local embeddings are then aligned and stitched into a single global coordinate frame via the embedding pipeline (e.g., synchronization, alignment, and refinement), after which the global node vectors $\{p_i\}$ are read off. After the sub-graphs are independently embedded (localized) and subsequently aligned through reflection, rotation, and translation, they are stitched together to form a consistent global embedding. Let the resulting aligned and stitched sub-graphs be denoted by $p_1, p_2, \ldots , p_n$, where *n* is the number of nodes across the entire network after alignment (note: $n = |V|$). Each $p_a \in \mathbb {R}^d$ is the estimated coordinate vector (typically in $\mathbb {R}^2$ or $\mathbb {R}^3$) of node *a* in the global coordinate frame.

The observed or reconstructed distance between any two nodes *a* and *j*, denoted $d_{aj}$, is then expressed as $d_{aj} = \Vert p_a - p_j\Vert + \varepsilon _{aj}$ where $\Vert p_a - p_j\Vert$ is the Euclidean distance between the estimated positions of nodes *a* and *j*, and $\varepsilon _{aj}$ represents the additive noise or distortion introduced during the sub-graph embedding and stitching process, due to misalignment, measurement error, or numerical approximation. The observed or reconstructed distance between any two nodes *a* and *j*, denoted $\bar{d}_{aj}$, is either (i) an observed measurement $d^{\textrm{obs}}_{aj} = r_{aj} + \eta _{aj}$ obtained from ranging or RSSI under measurement noise $\eta _{aj}$, or (ii) a reconstructed distance $d^{\textrm{rec}}_{aj} = \Vert \hat{x}_a - \hat{x}_j \Vert _2$ computed from the current embedding when a direct observation is unavailable or unreliable. Unless stated otherwise, references to “distance” use $\bar{d}_{aj}$, and the distance dataset is $\bar{\mathcal {D}} = \{\bar{d}_{aj}: (a,j) \in V \times V\}$.. It is worth-mentioning here that $P_a$ consists of all the nodes within 1-hop neighborhood of the *a*th IoT end-node. To analyze interactions between two nodes, we can find the intersection of their 1-hop neighborhoods. This intersection forms the vertex set of the subgraph *G*(*a*, *j*), where $(a,j) \in E(G(a,j))$ is an edge in the original graph. So considering the nodes *a* and *j*, let us assume that we want to align the sub-graphs $P_a$ and $P_j$. In order to achieve that, the first step is to formulate the relative reflection, $z_{aj} \in \{-1,+1\}$.

#### Sub-graph reflection

In large-scale IoT deployments, sub-graphs reconstructed from partial and noisy distance measurements may appear mirrored, rotated, or shifted with respect to one another due to measurement errors and independent initialization of local coordinate frames. Without proper alignment, identical spatial relationships across sub-graphs may map to inconsistent global positions, leading to unrealistic network geometries and distorted connectivity. To address this issue, IoTNTop performs a three-stage geometric synchronization procedure—reflection, rotation, and translation—to register all sub-graphs within a common coordinate system. This process ensures that the recovered spatial topology faithfully represents the underlying communication structure, thereby enabling reliable subsequent topology control and power–rate optimization.

We first construct an $N \times N$ sparse, symmetric *reflection-consistency matrix* $\mathcal {Z}^{\textrm{ref}} = (z^{\textrm{ref}}_{aj})$, defined as

3 $$\begin{aligned} \mathcal {Z}^{\text {ref}} = {\left\{ \begin{array}{ll} \;\;1 & \text {no reflection required for alignment}, \\ -1 & \text {reflection required}, \\ \;\;0 & \text {alignment not feasible}. \end{array}\right. } \end{aligned}$$

Each entry $z^{\textrm{ref}}_{aj} \in \{-1,0,+1\}$ encodes whether sub-graphs $P_a$ and $P_j$ require a reflection to be aligned based on their shared distance information.

Let $\Delta$ denote the diagonal degree matrix associated with $\mathcal {Z}^{\textrm{ref}}$, where each diagonal entry is defined as $\Delta _{\alpha \alpha } = \deg (\alpha ),$ with $\deg (\alpha )$ representing the number of sub-graphs that share sufficient landmarks with sub-graph $\alpha$ to permit alignment (i.e., non-zero entries in row $\alpha$ of $\mathcal {Z}^{\textrm{ref}}$). Using this degree matrix, we form the random-walk normalized reflection matrix

$$\zeta = \Delta ^{-1}\mathcal {Z}^{\textrm{ref}}.$$

The matrix $\zeta$ is used exclusively for eigenvector synchronization over the reflection group $\{-1,+1\}$ and is conceptually unrelated to coverage metrics or optimization variables introduced elsewhere in the paper.

We compute the dominant eigenvector $v^{\zeta }_1$ of $\zeta$, $\zeta v^{\zeta }_1 = \lambda ^{\zeta }_1 v^{\zeta }_1,$ and estimate the global reflection state of each sub-graph as

4 $$\begin{aligned} \hat{z}_a = \textrm{sign}\!\left( v^{\zeta }_1(a)\right) = \frac{v^{\zeta }_1(a)}{|v^{\zeta }_1(a)|}. \end{aligned}$$

If $\hat{z}_a = -1$, the corresponding sub-graph $P_a$ is reflected to its mirrored configuration prior to subsequent rotation and translation alignment steps.

#### Construction of $\mathcal {Z}^{\textrm{ref}}$

The reflection-consistency matrix $\mathcal {Z}^{\textrm{ref}}$ is constructed as follows. We initialize an $N \times N$ matrix with zero entries. For each pair of sub-graphs $P_a$ and $P_j$, we compute the Pearson correlation coefficient between their distance measurements over the set of shared nodes:

5 $$\begin{aligned} \tilde{r} = \frac{\sum _{i=1}^{n} (d_{ai} - \mu _a)(d_{ji} - \mu _j)}{(n - 1)\alpha _a \alpha _j}, \end{aligned}$$

where $\tilde{r}$ quantifies the linear correlation between the distance profiles of the two sub-graphs; $d_{ai}$ and $d_{ji}$ denote the Euclidean distance measurements from nodes *a* and *j* to the *i*th shared node; $\mu _a$ and $\mu _j$ are the corresponding sample means; $\alpha _a$ and $\alpha _j$ are the standard deviations of the distance measurements within sub-graphs $P_a$ and $P_j$, respectively; and *n* is the number of shared nodes.

If $\tilde{r} < r_{\textrm{thr}}$, with threshold $r_{\textrm{thr}} = -0.5$, we set $z^{\textrm{ref}}_{aj} = -1$, indicating that a reflection is required. Otherwise, we set $z^{\textrm{ref}}_{aj} = +1$. When insufficient overlap or unreliable measurements prevent meaningful correlation estimation, the entry is left as $z^{\textrm{ref}}_{aj} = 0$, indicating that alignment between the sub-graphs is not feasible and that the pair is excluded from synchronization. The threshold $r_{\textrm{thr}} = -0.5$ was selected empirically through a sensitivity analysis on simulated IoT networks with $N=100$ nodes and noise levels ranging from $20\%$ to $70\%$. The threshold was varied between $-0.3$ and $-0.7$ in increments of 0.1, and alignment accuracy was evaluated over 50 Monte-Carlo runs. The chosen value provided the best balance between false reflection detection and missed alignments, yielding consistent alignment accuracy within $\pm 2\%$ across all tested noise regimes.

#### Sub-graph rotation

We construct the $N \times N$ Hermitian matrix $R = (r_{aj})$ such that,

6 $$\begin{aligned} r_{aj} = {\left\{ \begin{array}{ll} e^{\imath \theta _{aj}} & \quad P_a~{ \& }~P_j~\text {can be aligned}\\ 0 & \quad P_a~{ \& }~P_j~\text {cannot be aligned} \end{array}\right. } \end{aligned}$$

where $\theta _{aj} \in [0,2\pi )$, $\theta _{aj} = - \theta _{ja} \mod 2\pi$, $r_{aj} = \bar{r}_{ja}$, for any complex number and its complex conjugate. We compute the top eigenvector $v_1^{\mathcal {R}}$ of $\mathcal {R} = \Delta ^{-1}R$ with $\mathcal {R} v^{\mathcal {R}}_1 = \lambda ^{\mathcal {R}}_1 v^{\mathcal {R}}_1$ and estimate,

7 $$\begin{aligned} e^{\imath \hat{\theta }_{a}} = v^{\mathcal {R}}_1(a)/|v^{\mathcal {R}}_1(a)| \end{aligned}$$

to rotate the patch $P_a$. The next step is to compute the translation of the sub-graph $P_b$ into aligned sub-graph $P_a$. Here $P_b$ is the *b*th patch or subgraph.

#### Construction of *R*

We start with an $N \times N$ matrix where initially all elements are 0. For each pair of subgraphs $P_a$ and $P_j$, where alignment is possible, we calculate $\theta _{aj}$ following the Landmark Matching procedure^26^. Let $\bar{X} = \{\bar{x}_1, \bar{x}_2, \cdots , \bar{x}_m\}$ with $\mu _{\bar{X}} = \frac{1}{m}\sum _{\bar{i} = 1}^m \bar{x}_{\bar{i}}$ as the mean points in $\bar{X}$, represent the coordinates of *m* shared landmarks in $P_a$, $\bar{Y} = \{\bar{y}_1, \bar{y}_2, \cdots , \bar{y}_m\}$, represent the corresponding coordinates in $P_j$ with $\mu _{\bar{Y}} = \frac{1}{m}\sum _{\bar{i} = 1}^m \bar{Y}_{\bar{i}}$ as the mean points in $\bar{Y}$, $\bar{z}_i = \bar{x}_i - \mu _{\bar{X}}$ is the zero-centered coordinate for landmark $\bar{i}$ in $P_a$, $\bar{w}_i = \bar{Y}_i - \mu _{\bar{Y}}$ is the zero-centered coordinate for landmark $\bar{i}$ in $P_j$, and $\textsf{H} = \sum _{\bar{i} = 1}^m \bar{z}_i \bar{w}_i$ is the covariance matrix between $\bar{X}$ and $\bar{Y}$.

Now let us define,

8 $$\begin{aligned} \text {Rotation :}~&\bar{y}_i \approx e^{\imath \theta } \bar{x}_i = (\cos \theta + \imath \sin \theta ) \bar{x}_i \end{aligned}$$

9 $$\begin{aligned} \text {Translation :}~&\bar{y}_i \approx e^{\imath \theta } \bar{x}_i + \bar{t}\end{aligned}$$

10 $$\begin{aligned} \text {Scaling :}~&\bar{y}_i \approx \bar{\textsf{s}}e^{\imath \theta } \bar{x}_i + \bar{t} \end{aligned}$$

where $\bar{t}$ is the translation vector and $\bar{\textsf{s}}$ is the scaling factor. The goal is to find the parameter values that minimize the discrepancy between the transformed landmarks from $P_a$ and their corresponding points in $P_j$. We formulate this as a least-squares problem;

11 $$\begin{aligned} \text {Minimize}_{\bar{\textsf{s}}, \bar{t}} :~\sum _{\bar{i} = 1}^m ||\bar{\textsf{s}}e^{\imath \theta } \bar{x}_i + \bar{t} - \bar{y}_i||^2 \end{aligned}$$

In order to remove the effect of global translation, we substitute $\bar{z}_i$ and $\bar{w}_i$ for the centered coordinates in (11) to obtain the optimization problem as,

12 $$\begin{aligned} \text {Minimize}_{\bar{\textsf{s}}, \bar{t}} :~\sum _{\bar{i} = 1}^m ||\bar{\textsf{s}}e^{\imath \theta } (\bar{x}_i - \mu _{\bar{X}}) + (\bar{t} - \mu _{\bar{Y}}) - (\bar{y}_i - \mu _{\bar{Y}})||^2 \end{aligned}$$

We solve the optimization problem using singular value decomposition (SVD) for the covariance matrix $\textsf{H}$, to extract the value of $\theta$ using trigonometric functions and identities and then solve for $e^{\imath \theta }$ as a function of eigenvectors and eigenvalues of $\textsf{H}\textsf{H}^T$. Optimality of (12) is detailed in Appendix A.

#### Sub-graph translation

For $P_b$, we compute,

13 $$\begin{aligned} p_a = p_a^{(b)} + t^{(b)}; i \in V_a; b = 1, \cdots , N \end{aligned}$$

where $t^{(b)}$ is the associated translation. Using the over-determined system of (13), we can estimate the global coordinates, $p_1, \cdots , p_n$ using least square solution to (13). The set of translations $t^{(1)}, \cdots , t^{(N)}$ is disregarded. So for each edge $(a,j) \in E_b$, $p_a - p_j = p_a^{(b)} - p_j^{(b)}$. The term $E_b$ refers to the set of edges within the $b$-th sub-graph, i.e., $E_b \subseteq E$, where $E$ is the overall edge set of the original IoT network graph. Each sub-graph $P_b$ contains a subset of nodes and the corresponding intra-subgraph links, and $E_b$ captures these localized connectivity relationships. When solving the over-determined system of equations to compute global node coordinates, we aggregate constraints from all edges $(a, j) \in E_b$ across sub-graphs, ensuring consistency and continuity in the global graph embedding. If we replace the patches with the $(\textsf{x},\textsf{y})$ coordinates, $\textsf{x}_a - \textsf{x}_j = \textsf{x}_a^{(b)} - \textsf{x}_j^{(b)}; \textsf{y}_a - \textsf{y}_j = \textsf{y}_a^{(b)} - \textsf{y}_j^{(b)}$, which can be solved separately.

Now we can write, $\tau \textsf{x} = \gamma ^{\textsf{x}}$, where $\tau$ is the least square translation matrix. It is constructed based on the relationships between the local coordinates of nodes in different subgraphs and their corresponding global coordinates. $\textsf{x}$ is the $n \times 1$ vector of $\textsf{x}$-coordinates of all IoT end-nodes localized in a sub-graph. The $n \times 1$ vector represents the $\textsf{x}$-coordinates of all the IoT end-nodes that have been localized within a particular subgraph. The goal is to estimate these global $\textsf{x}$-coordinates. $\gamma ^{\textsf{x}}$ is the vector with entries from the right-hand side of (13). The vector containing the right-hand side values of the linear equations is derived from the subgraph translation process. These values are based on the local coordinates and the estimated translations between subgraphs. Similarly, we can write $\tau \textsf{y} = \gamma ^{\textsf{y}}$. The accuracy of these estimated coordinates is then evaluated by comparing them to the true coordinates using an error metric. Now by adding all the equations corresponding to the same edge (*a*, *j*) from different patches,

14 $$\begin{aligned}&\sum _{k \in \{1, \cdots , N\}; (a,j) \in E_b} \textsf{x}_a - \textsf{x}_j \nonumber \\&= \sum _{k \in \{1, \cdots , N\}; (a,j) \in E_b} \textsf{x}_a^{(b)} - \textsf{x}_j^{(b)},~(a,j) \in E \end{aligned}$$

and similarly for the $\textsf{y}$-coordinates we can formulate the least-square translation matrix $\tau$, i.e. $m \times n$ over-determined system of linear equations. If the estimated transition matrix is denoted by $\hat{\tau }$, the least square solutions to $\hat{\tau }\textsf{x} = \gamma ^{\textsf{x}}$ and $\hat{\tau }\textsf{y} = \gamma ^{\textsf{y}}$ will be given by, $\hat{p}_1, \cdots , \hat{p}_n$. The error in any estimated sub-graph coordinates can be calculated as $||p_a - \hat{p}_a||$. Now that we have solved the localization problem for IoT end-nodes, we can find the union of set $\mathcal {L}$ (end-nodes) and set $\mathcal {S}$ (gateways) to determine the locations of all nodes within the network.

The proposed alignment framework assumes moderate overlap between adjacent sub-graphs; however, in practice, link sparsity, high noise, or missing landmarks can degrade synchronization quality. To address this, IoTNTop employs two adaptive mechanisms: (i) a *connectivity-aware expansion* that temporarily enlarges low-overlap sub-graphs using nearest-neighbor augmentation until a minimum landmark ratio is met; and (ii) a *noise-weighted confidence score* that down-weights distance pairs with high variance during EVS computation, reducing the impact of unreliable edges. When landmark information is insufficient for global stitching, the system reverts to localized EVS alignment within connected components until new measurements become available. These mechanisms improve stability and maintain feasible embeddings even under adverse sensing conditions.

### Graph-embedding for central and end-nodes

Let us assume that we want to connect the *i*th gateway, $i = 1, \cdots , s$ with the *j*th end-node, $j = 1, \cdots , l$. Therefore the Euclidean distance is $d_{ij}$ between the *i*th gateway and the *j*th end-node, where $(i,j) \in \mathcal {S} \cup \mathcal {L}$ and $\bar{E}(\mathcal {L}, \mathcal {L})$ are the set of edges between end-node and gateway, and two end-nodes, respectively. We now form a new network graph, $\bar{G} = (\bar{V},\bar{E})$ with $\bar{V} = \mathcal {S} \cup \mathcal {L}$ of size $|\bar{V}| = l + s$ and edge set $|\bar{E}| = m + \bar{m}$ where $\bar{m}$ is the number of edges between the end-nodes and gateways of the network. Here, we introduce the augmented edge set $\bar{E}$, which extends the original candidate edge set $E$ by including both end-node–to–end-node links and end-node–to–gateway links after quality gating. Specifically, $E$ represents the initial set of feasible communication links inferred from the SNR and distance thresholds among end-nodes only, while $\bar{E} = E \cup E_{(L,S)}$ augments this set by adding the cross-layer connections between end-nodes ($L$) and gateways ($S$) that satisfy the decoding and signal-quality criteria. In cases where link reliability falls below the SNR threshold or distance constraints are violated, the corresponding edges are pruned from $\bar{E}$. Thus, $\bar{E}$ serves as the refined, quality-gated edge set used in the joint embedding and topology-control stages, ensuring that only valid, high-quality links contribute to the construction of the global graph $\bar{G}$.

The partial distance measurement matrix is given by, ${\bf D} = \{d_{ij}:(i,j)\in \bar{E}(\mathcal {L}, \mathcal {L}) \cup \bar{E}(\mathcal {L}, \mathcal {S})\}$ and the graph realization problem (in this work, “realization” refers to graph realization or localization from partial and noisy pairwise distance information, producing a globally consistent geometry (node positions for gateways and end-nodes) that respects local measurements. Our pipeline links topology control and realization as follows: topology control selects feasible links and power/rate settings under reliability and energy constraints; the resulting measurement set and link feasibility inform the realization step, which stitches local sub-graphs into a single geometry. This geometry, in turn, feeds back into the next topology-control epoch by updating path-loss, SNR, and code-rate feasibility, ensuring that connectivity decisions remain aligned with the recovered spatial structure) becomes,

15 $$\begin{aligned} ||\textsf{x}_i - \textsf{x}_j||^2_2&= d^2_{ij}~\text {for}~(i,j) \in \bar{E}(\mathcal {L},\mathcal {S}) \nonumber \\ ||\textsf{x}_j - \textsf{x}_a||^2_2&= d^2_{ja}~\text {for}~(j,a) \in \bar{E}(\mathcal {S},\mathcal {S}) \nonumber \\&\text {for}~i = 1, \cdots , s, j = 1, \cdots , l, a = 1, \cdots , l. \end{aligned}$$

The symbol $E_{(L,S)}$ denotes the subset of edges connecting end-nodes in the set $L$ to gateways in the set $S$. Each edge $(i,j) \in E_{(L,S)}$ represents a feasible end-node–to–gateway communication link that satisfies the SNR and distance constraints. Together with the end-node–to–end-node links $E_{(L,L)}$, these form the composite edge set used to construct the augmented network graph $\bar{G} = (\bar{V}, \bar{E})$, where $\bar{E} = E_{(L,L)} \cup E_{(L,S)}$. This distinction allows the model to treat intra-cluster (L–L) and cross-cluster (L–S) communications separately when embedding and optimizing the topology.

We start by randomizing the realization of the sub-graph embeddings $p_1, \cdots , p_n$. We initialize the unknown global coordinates $\{p_i\}_{i=1}^{n}$ by placing each locally embedded patch into the global frame with a random rigid motion, a uniformly sampled rotation $\theta \in [0,2\pi )$, an optional reflection, and a small random translation. This produces a non-degenerate warm start that breaks symmetries and avoids coincident placements. The subsequent EVS and LA synchronization and SDP refinement remove the dependence on this random initialization and determine the final, globally consistent embedding. We also introduce a small positive constant of $\varrho$ (for example, $\varrho = 10^{-5}$), small enough to filter out noisy or unstable positions, but not so small that it excludes nearby correct embeddings due to floating-point precision issues. Once we have the end-node network mapping in place, we look at the network between the end-nodes and gateways by solving the SDP problem,

16 $$\begin{aligned} \text {Maximize}~0,~\rightarrow ~\text {subject to},~({\bf 0}; e_i-e_j)({\bf 0};e_i-e_j)^T \chi = d^2_{ij} \end{aligned}$$

for $(i,j) \in \bar{E}(\mathcal {L},\mathcal {S})$ and $\mu \in \kappa ^{l + s}$ where $e_i$ is an all-zeros vector with *i*th entry of 1 and

17 $$\begin{aligned} \kappa ^{l + s} = \Bigg \{\chi _{(l+s)\times (l+s)}~\Bigg |~\chi = \begin{bmatrix} I_s & \textsf{X}\\ \textsf{X}^T & \textsf{Y} \end{bmatrix} \ge 0\Bigg \} \end{aligned}$$

where $\chi$ is the matrix representation of $(\textsf{X},\textsf{Y})$ coordinates. If the vector $\omega$ represents the diagonal elements of the matrix $\textsf{Y} - \textsf{X}\textsf{X}^T$, we compute the new subset of vertices $\tilde{V} \in \bar{V}\backslash \mathcal {S}$ such that $\omega _i < \varrho$ and form the end-node -gateway sub-graph, $\tilde{G} = (\tilde{V}, \tilde{E})$. We formulate the solution to (16) as SDP matrices, as eigenvalues being non-negative means the resulting computed ‘distances’ will make physical sense and SDP matrices generally enforce the triangle inequality, which is essential for realistic distances. For each candidate link $(i,j)$, link quality is quantified by the instantaneous SNR $\textrm{SNR}_{ij}$ defined in the previous section. A link is retained in $\bar{E}$ if $\textrm{SNR}_{ij}\ge \textrm{SNR}_{\textrm{thr}}$ and its achievable code rate $R_{ij}$ is feasible under the chosen coding profile, otherwise it is pruned.

In (16), $\text {Maximize}~0$ indicates that the optimization problem aims to maximize the objective function while ensuring that all constraints are satisfied. In (16), the optimizing variable is the matrix $\chi \in \mathbb {R}^{(l+s) \times (l+s)}$, which represents the embedding of both end-nodes and gateways in the combined coordinate space. Specifically, $\chi$ is structured as a block matrix containing the coordinate relationships between nodes, where the diagonal and off-diagonal entries encode squared distances and inner products derived from the estimated node positions. The optimization seeks a feasible $\chi$ that satisfies the set of distance constraints $(0; e_i - e_j)(0; e_i - e_j)^T \chi = d_{ij}^2$ for all pairs $(i, j) \in \bar{E}(L, S)$, while also ensuring that $\chi$ remains positive semi-definite. Solving this problem yields a coordinate embedding consistent with available partial distance measurements and the underlying network connectivity. The ’0’ in this context is a placeholder or represent a null objective function, implying that the primary focus is on feasibility rather than optimizing a specific numerical value. The actual optimization occurs through the constraints, which enforce conditions on the variables involved. Optimality of (16) is detailed in Appendix B.

#### Insights into $\chi$

$\chi$ is a block matrix representation of the coordinates of both end-nodes and gateways. It has dimensions $(l + s) \times (l + s)$. Here $I_s$ corresponds to fixed gateway node positions and is an $s\times s$ identity matrix, $\textsf{X}$ is an $s \times l$ matrix where each column will hold the coordinates of an end-node to be estimated, $\textsf{X}^T$ is the transpose of the end-node coordinate matrix, and $\textsf{Y}$ is the $l \times l$ matrix where the diagonal elements will hold the squared pairwise distances between the end-nodes while the off-diagonal elements will be filled during the solution process.

Let us consider a small network with 2 gateways and 3 end-nodes. Here $\chi$ will look like,

18 $$\begin{aligned} \chi = \begin{bmatrix} 1 & 0 & \textsf{x}_1 & \textsf{x}_2 & \textsf{x}_3\\ 0 & 1 & \textsf{y}_1 & \textsf{y}_2 & \textsf{y}_3\\ \textsf{x}_1 & \textsf{y}_1 & \cdot & \cdot & \cdot \\ \textsf{x}_2 & \textsf{y}_2 & \cdot & \cdot & \cdot \\ \textsf{x}_3 & \textsf{y}_3 & \cdot & \cdot & \cdot \end{bmatrix} \end{aligned}$$

The SDP formulation, together with specific structure of $\chi$, allows to optimize the coordinates of the end-nodes to best match with the available set of distance measurements. Crucially, our formulated method assumes that we don’t have direct pairwise distance measurements for all possible node pairs–a realistic and desirable design feature for large-scale IoT networks. In practical deployments, obtaining complete distance information is infeasible due to energy constraints, communication range limitations, and environmental noise. By explicitly working with partial and noisy measurements, our approach remains scalable, resilient to missing data, and well-suited for resource-constrained and dynamically evolving network topologies.

**Fig. 2:**
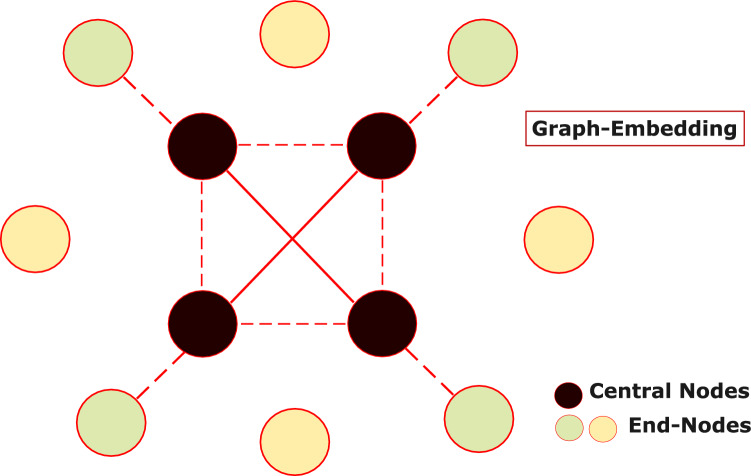
Representation of relationship between Central and End-Nodes under optimized topology control. This figure depicts how IoTNTop uses partial, noisy pairwise distances $D$ together with node roles (end-nodes $L$ and gateways $S$) to construct the augmented graph $\bar{G}=(\bar{V},\bar{E})$ via EVS/LA stitching and SDP refinement, applies link-quality gating on the composite edge set $\bar{E}=E_{(L,L)}\cup E_{(L,S)}$ using per-link SNR and code-rate feasibility, and outputs a reliability-aware topology that activates only high-quality $L\text {--}S$ and necessary $L\text {--}L$ links for robust coverage, low error probability, and energy-efficient operation.

Additionally, we assume that the network is connected—or, at a minimum, consists of connected components that are sufficiently large to allow meaningful embedding. This assumption is important because graph embedding and localization techniques rely on relative distance information between nodes. If the network is fragmented into very small or isolated components, there won’t be enough pairwise measurements to accurately determine the spatial relationships among nodes. Ensuring basic connectivity allows our algorithm to propagate positional information across the network and produce coherent global topologies. Finally, the SDP formulation is more robust to noisy measurements by adding regularization terms or relaxing the equality constraints in (16).A conceptual overview of the relationship between central and end-nodes in provided in Fig. 2. Figure 2 connects geometric embedding (recovering the global layout from partial noisy distances) with reliability-driven topology control (activating only links that meet decoding feasibility), making explicit how the method reduces symbol-error probability while respecting per-node power budgets.

### Sub-graph embedding for the global graph

For sub-graph embedding in our globally non-rigid IoT network graph, we resort to the three-step method outlined in^6^ (it is worth-mentioning here, that the term ’rigid’ refers to the property of a graph or network where the relative distances between all nodes remain constant. In other words, a rigid graph maintains its shape and structure even when subjected to transformations like rotations or translations. In this work, we resort to a non-rigid approach for sub-graph embedding because we are looking at topology optimization of IoT networks in a dynamic scenario). The first step is to estimate the distance, $d'_{ij}$ with $(i,j) \notin E_k$ (we are considering an annular graph, with a network where the IoT devices are distributed randomly on an annulus surrounding the IoT gateway at the center). $d'_{ij} = (\underline{d_{ij}} + \overline{d_{ij}})/2$, for,

19 $$\begin{aligned} \overline{d_{ij}}&= (\overline{d_{aj}} + \overline{d_{ai}})/2; \nonumber \\ \overline{d_{aj}}&= \min _{k:(a,k),(j,k) \in E_k} d_{ak} + d_{jk};~\overline{d_{ai}} \nonumber \\&= \min _{k:(a,k),(i,k) \in E_k} d_{ak} + d_{ik}\end{aligned}$$

20 $$\begin{aligned} \underline{d_{ij}}&= \max \bigg [\max _{k:(a,k)\in E_k}d_{ak}, \max _{k:(i,k)\in E_k}d_{ik}, \max _{k:(j,k)\in E_k}d_{jk}\bigg ] \end{aligned}$$

The second step is to compute local coordinates of all nodes in a sub-graph using multi-dimensional scaling^17^ on the complete set of pairwise distances. We can express, ${\bf D} = -1/2 {\bf J}{\bf L}{\bf J}$ where ${\bf J} = {\bf I}_{s+l} - 1/(s+l) {\bf 1}{\bf 1}^t$, ${\bf 1}$ is a matrix of ones, ${\bf I}$ is the identity matrix, ${\bf L}$ is the matrix of squared pairwise distances, ${\bf L} \in \mathbb {R}^{(s+l) \times (s+l)}$, *t* denotes the transpose, $s+l \ge 1$ is an integer, and the matrix of the local coordinates of all nodes in a patch can be calculated as,

21 $$\begin{aligned}&P_k ={\bf U}_k \sqrt{\Lambda _k} \nonumber \\&= \bigg [\sqrt{\lambda _1}{\bf u}_1, \cdots , \underbrace{\sqrt{\lambda _{f+1}}{\bf u}_{f+1}}_{= 0}, \cdots , \underbrace{\sqrt{\lambda _k}{\bf u}_k}_{= 0}\bigg ] \in \mathbb {R}^{(s+l) \times k} \end{aligned}$$

where $({\lambda _{(s+l)}},{\bf u}_{(s+l)})$ are the eigen-pairs of the ${\bf D}$ matrix.

The final step is to refine the embedding using iterative majorization technique. The coordinates of each IoT node are updated according to,

22 $$\begin{aligned} p_i \leftarrow 1/~\text {deg}_i(P_k)&\sum _{j \in V_k, (a,i,j) \in E_k} [p_j + d_{ij}(p_j - p_a)\nonumber \\&\times \text {inv}~(||p_j - p_a||)] \end{aligned}$$

where $\text {deg}_i(P_k)$ denotes the degree of node *i* in patch $P_k$ and,

$$\text {inv}~(\textsf{x}) = {\left\{ \begin{array}{ll} 1/\textsf{x} & \quad \text {if } \textsf{x} \ne 0\\ 0 & \quad \text {if } \textsf{x} = 0 \end{array}\right. }$$

**Fig. 3:**
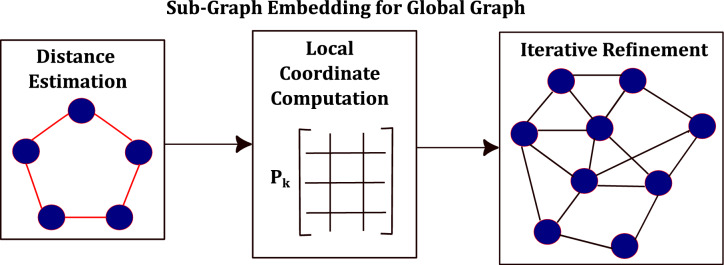
IoTNTop epoch: EVS and LA stitching $\rightarrow$ SDP refinement $\rightarrow$ SNR-guided edge selection $\rightarrow$ convergence check on global error & code rate.

A conceptual overview of sub-graph embedding in provided in Fig. 3.

### Aligning sub-graphs and sewing them together

Now after finding the embedding for the sub-graph $P_k$, we need to combine under the same objective function, the contribution of the end-node-to-end-node communications and the end-node-to-gateway communications. For the above, we formulate a least-square-problem-based synchronization as,

23 $$\begin{aligned} \min _{\textsf{x}} \sum _{(i,j)\in \bar{E}} (\textsf{x}_i - \chi _{ij}\textsf{x}_j)^2 = \min _{\textsf{x}}~{\textsf{x}}^T ({\bf D} - \chi )\textsf{x} \end{aligned}$$

where,

24 $$\begin{aligned} \chi = \begin{bmatrix} \mathcal {\tilde{L}} & \mathcal {\tilde{S}}\\ \mathcal {\tilde{S}}^T & \mathcal {W} \end{bmatrix},~ {\bf D} = \begin{bmatrix} {\bf D}_{\mathcal {\tilde{L}}} & 0\\ 0 & {\bf D}_{\mathcal {W}} \end{bmatrix} \end{aligned}$$

where ${\bf D}_{\mathcal {\tilde{L}}}$ is the pairwise distance matrix within the set $\mathcal {L}$, ${\bf D}_{\mathcal {W}}$ is the pairwise distance matrix within the set $\mathcal {W}$. The sets, $\mathcal {\tilde{L}}_{c \times c}$, $\mathcal {\tilde{S}}_{c \times k}$ and $\mathcal {W}_{k \times k}$ denote the end-node-to-end-node, end-node-to-gateway and gateway-to-gateway measurements. We will use the Lagrangian multiplier $\mu$ concept to solve the minimization problem of (23) as,

25 $$\begin{aligned} \chi ^* = \big ({\bf D}_{\mathcal {\tilde{L}}} - \mathcal {\tilde{L}} + \mu {\bf I}\big )^{-1}(\tilde{\mathcal {S}}\sigma ) \end{aligned}$$

where $\sigma$ denotes the sign of the gateway vertices, +-ve sign represents similar or cooperative vertices (gateway vertices that have some degree of functional redundancy or complementary roles within the network), −-ve sign represents dissimilar or competitive vertices (gateway vertices that have conflicting roles or compete for resources within the network). In Algorithm 1, we summarize our proposed algorithm for graph embedding within a massive IoT network with multiple heterogeneous IoT nodes and more than one central node or gateway.

Let us consider the true distance measurement between the *j*th and *a*th subgraphs as $\delta _{aj} = ||p_j - p_a||$ with random noise of $\epsilon _{aj}$ added to the distance measurement distributed uniformly over the range $[-\eta \delta _{aj}, \eta \delta _{aj}]$. Consequently, we can express the noisy distance measurements as $d_{aj} = \delta _{aj} + \epsilon _{aj}$. We consider networks of size 20, 50 and 100 nodes with average node degree of 14 - 20 and noise levels up to 70% ($\eta = 0.7$ corresponding to 70%). We consider a network with gateways within a range of $\tilde{\rho }$ of different subgraphs. Let the true coordinates of all the nodes within subgraphs be represented by a $2 \times n$ matrix, $P = (p_1, \cdots , p_n)$ with estimated coordinates of $\hat{P} = (\hat{p}_1, \cdots , \hat{p}_n)$. We can compute the error in global localization of the nodes and the gateway owing to noisy measurement as,

26 $$\begin{aligned} \mathcal {A}_{e} = \frac{\sqrt{\sum _{j = 1}^n ||p_j - \hat{p}_j||^2}}{\sqrt{\sum _{j = 1}^n ||p_j - p_0||^2}} = \frac{||P - \hat{P}||_F}{||P - p_0{\bf 1}^t||_F} \end{aligned}$$

where $||\cdot ||_F$ denotes the Frobenius norm, *t* denotes transpose, ${\bf 1}$ denotes matrix containing all ‘1’ elements, and $p_0 = \frac{1}{n}\sum _{j =1}^n p_j$ represents the center of mass of all the true coordinates of the subgraphs. The denominator in (26) is the scaling and normalization factor for the graph embedding from the network perspective, representing the ‘spread’ of the true node coordinates around their center of mass. This makes the error metric less sensitive to mere scaling differences. Both the numerator and denominator include a subtraction of the center of mass ($p_0$). This makes the metric largely insensitive to uniform translations of the entire embedding. By factoring out scale and translation, (26) concentrates on measuring the error in the relative placement of nodes, which is often the key goal in graph embedding tasks.

The primary goal of graph embedding is to capture the relationships between nodes based on their relative distances and positions, with less emphasis on the absolute location of the entire graph. By subtracting the center of mass, the focus shifts to errors in relative node placement rather than overall graph positioning. Shifting the entire set of estimated coordinates ($\hat{P}$) should not significantly affect the error metric ($\mathcal {A}_{e}$). Aligning both the true (*P*) and estimated ($\hat{P}$) coordinates with their respective centers ensures that the metric emphasizes relative positioning over absolute location.

**Fig. 4:**
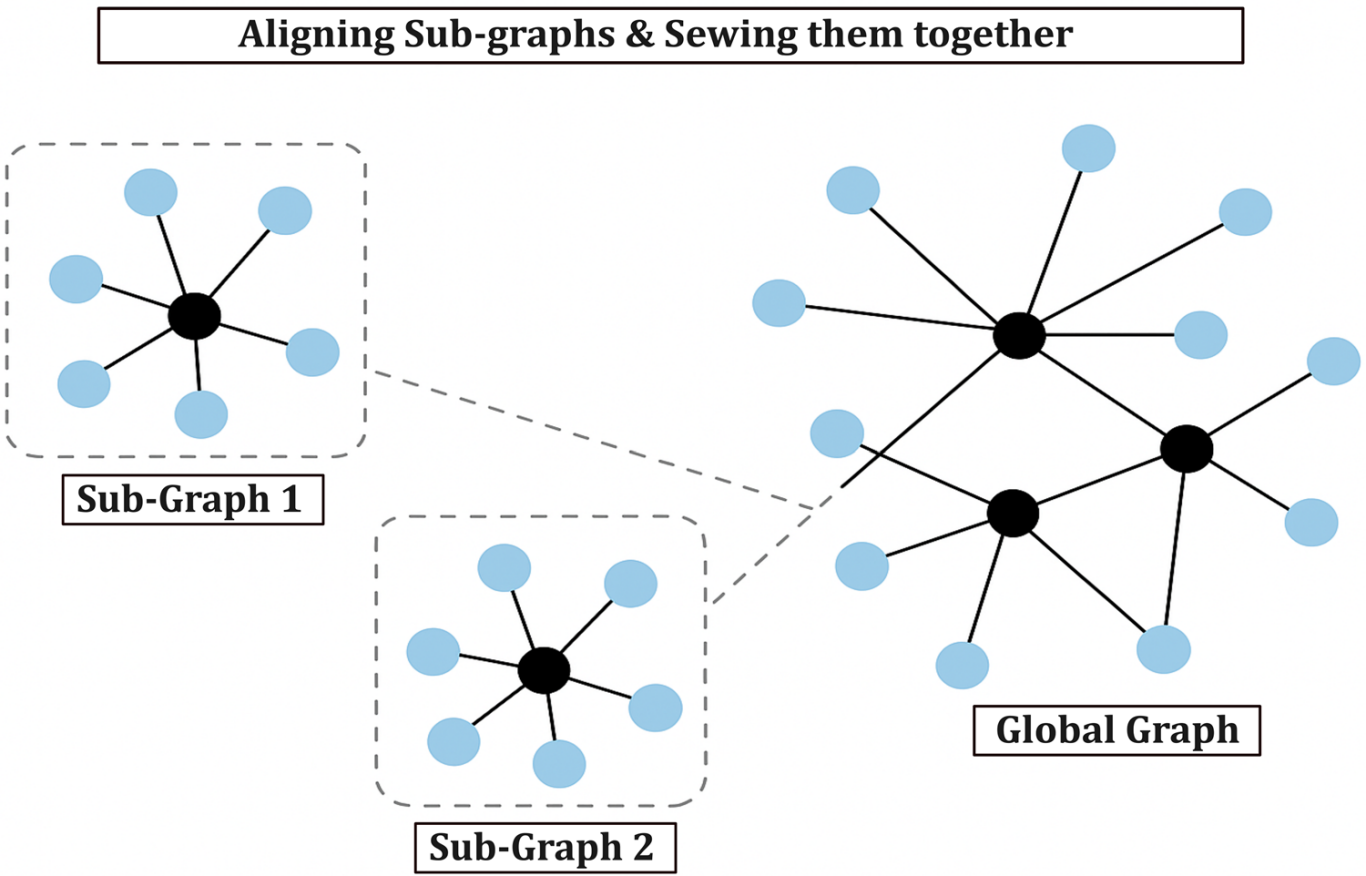
Aligning and sewing local sub-graphs into a globally consistent network. Two locally embedded sub-graphs (Sub-Graph 1 and Sub-Graph 2), each obtained from partial and noisy distance measurements, are aligned and stitched together through geometric transformations to form a unified global graph. This process resolves local ambiguities and enables consistent topology control at the network level.

In summary, to form a globally consistent topology from locally embedded sub-graphs, our approach performs sub-graph stitching through a three-stage alignment process involving reflection, rotation, and translation. First, we detect whether sub-graphs require reflection by computing the Pearson correlation coefficient between shared node distances; this information is used to construct a reflection matrix $Z$, from which global reflection decisions are derived via eigenvector synchronization ((1)–(2)). Second, we compute the relative rotation angle $\theta _{aj}$ between sub-graphs $P_a$ and $P_j$ by aligning their common landmarks using Procrustes analysis and SVD, resulting in a Hermitian matrix $R$ (4), from which global rotation estimates are extracted via eigenvector decomposition. Third, we estimate sub-graph translations using a set of linear equations formed from overlapping nodes, expressed as $\tau _x = \gamma _x$ and solved using least squares minimization ((11)–(12)). After reflection and rotation have been applied to each sub-graph, these translations align their coordinates in a common global space. Finally, we perform a least-squares synchronization over the composite graph Laplacian to refine the global topology (21). This layered approach enables us to consistently and efficiently embed sub-graphs with noisy and incomplete distance information, producing a coherent global graph suitable for topology control. A conceptual overview of aligning and sewing in provided in Fig. 4. Figure 4 provides a visual walkthrough of the embedding and control pipeline used within each epoch.

## Topology design and control

Next we formulate the topology extraction for an IoT network as an optimization problem with the target of minimizing error probability and maximizing information transmission rate over the link between the IoT nodes and the network gateways (refer to Algorithm 1). In the proposed mathematical model, we also consider (i) the transmit power from the node’s side and received power at the gateway end, (ii) how much energy is consumed when an IoT node fires its signal with a certain set of symbol coding on the information (the symbol coding can be simple space-time coding or other more complicated coding techniques like turbo coding) and (iii) how much energy is consumed on the gateway side for absorbing the received signal. We also consider the link quality as a defining factor using the pathloss exponent, the transmission range of the node, and the far-field reception range for the gateway. However, we do not consider the energy consumption for accumulating and computing information at the IoT nodes in our problem formulation.

We formulate and analyze a joint optimization over code rate, transmit power, and edge activation that minimizes network-level E2E error probability under power and decoding-feasibility constraints. This error-centric formulation, coupled with EVS, LA and SDP embedding and an SNR-guided convergence-checked solver, constitutes the core theoretical advance of *IoTNTop*.

### Information coding for transmission

Let us consider the $j$-th IoT node communicating with the $i$-th gateway separated by Euclidean distance $d_{ji}$. Let the $j$-th node transmit with power $P_T(j)$ and use an encoding function $\textsf{f}(j)$ to generate a sequence of $\bar{n}$ input symbols from random variables, $\tilde{\mathbb {W}} = \textsf{f}(\tilde{\mathcal {X}}^j_1, \cdots , \tilde{\mathcal {X}}^j_{\bar{\textsf{n}}})$ is the input on the link between (*j*, *i*). For the *i*th gateway, let the $\bar{\textsf{k}}$th received symbol be $\tilde{\mathcal {Y}}^i_{\bar{\textsf{k}}}$, so we can write,

27 $$\begin{aligned} \text {Pr}\{\tilde{\mathcal {Y}}^i_{\bar{\textsf{k}}} = \tilde{\textsf{y}}|\tilde{\mathcal {X}}^j_{\bar{\textsf{k}}} = \tilde{\textsf{x}}, \varepsilon _{\bar{\textsf{k}}}\} = \mathbb {Q}_{ji}(\tilde{\textsf{y}}|\tilde{\textsf{x}}) \end{aligned}$$

where $\tilde{\mathcal {X}}$ represents the input alphabet and $\tilde{\mathcal {Y}}$ represents the output alphabet of the links between the IoT end-nodes and gateways, $\mathbb {Q}$ is used to represent the actual link as a transfer function between the input and the output, and $\varepsilon _{\bar{\textsf{k}}}$ is a set of random variables. This objective aligns topology selection with decoding reliability by minimizing the network-level E2E error probability while respecting code-rate and power budgets. It is the global quantity IoTNTop seeks to reduce.

Unless otherwise noted, we adopt an error-prioritizing constraint form. We minimize network-level E2E error while enforcing a code-rate floor consistent with the selected coding profile and per-device power limits. Concretely, the optimization is solved in error constraint mode (error as the primary objective; code rate as a hard constraint), which operationalizes our claim of prioritizing error. We encode prioritization via a single weighted objective that balances network-level E2E error against achievable code rate. Let $\lambda \in [0,1]$ denote the trade-off weight; larger $\lambda$ places more emphasis on minimizing error, and smaller $\lambda$ places more emphasis on maximizing rate. In all main simulations, we use $\lambda =\boldsymbol{\lambda _0}$ (chosen to match the coding profile’s operating region and validated by sensitivity checks), and we keep all power and feasibility constraints unchanged.

**Algorithm 1:**
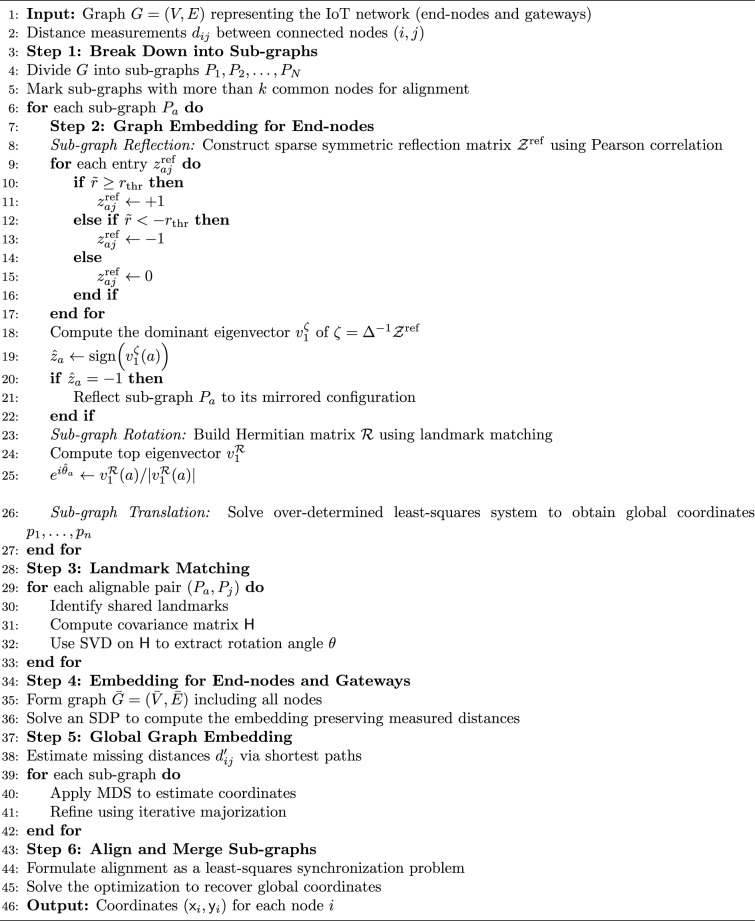
IoTNTop-graph alignment algorithm.

Let the decoding function of the *i*th gateway be given by $\textsf{g}$ and the recovered message be of the form $\tilde{\mathbb {W}}'$ denoted by, $\tilde{\mathbb {W}}' = \textsf{g}(\tilde{\mathcal {Y}}^i_1, \cdots , \tilde{\mathcal {Y}}^i_{\bar{\textsf{n}}})$, where $\tilde{\mathbb {W}}'$ is also a random variable of length $\bar{\textsf{m}}$. Then the error probability performance of the link is given by,

28 $$\begin{aligned} \Phi = \max _{\tilde{\mathbb {W}} \in \{1, \cdots , \bar{\textsf{m}}\}} \text {Pr}\{\tilde{\mathbb {W}}' \ne \tilde{\mathbb {W}}\} \end{aligned}$$

where, $\Phi$ is the maximum probability of error in recovering the original message $\tilde{\mathbb {W}}$, considering all possible original messages within the set $\{1, \cdots , \bar{\textsf{m}}\}$ and a coding rate ${\mathcal {R}}$ is achievable if there exists a sequence of $(\lceil 2^{\bar{\textsf{n}},\mathcal {R}}\rceil , \bar{\textsf{n}})$ codes such that $\Phi$ tends to 0 as $\bar{\textsf{n}}$ becomes infinitely large. In turn, we can also calculate the capacity of the link which is the supremum of all the achievable rates. This constraint ties the achievable code rate to link SNR and pathloss, ensuring that selected links operate inside the feasible decoding region for the chosen coding profile.

The greedy SNR-based optimization occupies the final stage of each epoch after geometric realization/embedding and before metric logging. Concretely, the epoch proceeds as: (a) acquire partial/noisy pairwise measurements and link-quality summaries; (b) update the geometric embedding using sub-graph stitching and refinement; (c) *greedy edge selection*: sort candidate links by current signal quality and code-rate feasibility, iteratively activate links that reduce the global error objective while respecting per-device power and capacity limits, and skip links that violate feasibility or offer no objective improvement; (d) *convergence check*: stop when global error and code-rate indicators change less than a small tolerance or a maximum iteration count is reached; (e) apply decisions by updating active links, per-node transmit powers, and link code rates for the next epoch.

### Transmit power allocation

Given the transmit power $P_T(j)$ of node $j$, the received power when node $j$ transmits can be expressed as,

29 $$\begin{aligned} \mathcal {P}_T(\textsf{r}_j) = \kappa _1 \textsf{r}^{\nu }_j + \kappa _2 \end{aligned}$$

where $\textsf{r}_j$ is the transmission range of the *j*th node, $\nu$ is the pathloss exponent, $\kappa _1$ is the constant defining the IoT node type, like whether it is a bi-static or a multi-static sensor, and $\kappa _2$ quantifies the link quality between the *j*th node and the *i*th gateway that accounts for non-distance-related factors affecting signal reliability, namely, fading, interference from neighboring devices, environmental noise, receiver sensitivity variations at the gateway and antenna alignment mismatch. This enforces per-node power limits (and, when aggregated, a network budget), preventing solutions that unrealistically increase transmit power to reduce error. Now on the gateway side, the received power can be calculated as,

30 $$\begin{aligned} \mathcal {P}^{ij}_R(d_{ji}) = \frac{\mathcal {P}^{ij}_R(d_{0i}) \times d^{\nu }_{0i}}{d^{\nu }_{ji}} \end{aligned}$$

where $\mathcal {P}^{ij}_R(d_{ji})$ is the received power at the *i*th gateway from the *j*th node over the Euclidean distance $d_{ji}$ and $\mathcal {P}^{ij}_R(d_{0i})$ is the received power at the *i*th gateway over the far-field distance $d_{0i}$. This provides an information-theoretic bound and approximation that maps SNR and code rate to symbol or packet error probability; it is how physical-layer reliability enters the optimization. Using the above formulations in (29) and (30), we can design the error probability minimization problem, with constraint on code rate maximization as,

31 $$\begin{aligned} \text {minimize}_{\mathbb {Q}_{ji}}~~\mathcal {G}(\Phi )&= \Bigg [\frac{\textsf{r}^{\nu }_j}{\mathcal {E}_j} + d^{\nu }_{ji}\Bigg ]\times \mathbb {Q}_{ji} \nonumber \\ \text {subject to,}~~&\mathbb {Q}_{ji} = \max _{a \in V} (\textsf{h}_{ja})~\text {and}~0 \le \textsf{h}_{ja} \le \mathcal {R}_{ja}\nonumber \\&\text {and}~0 < \textsf{r}_j \le \textsf{r}_{\text {max}}\nonumber \\&\sum _{a|a \in V} \textsf{h}_{ja} - \sum _{a|j \in V} \textsf{h}_{aj} = \psi _j~\text {for}~d_{ji} \le \textsf{r}_j \end{aligned}$$

where $\mathcal {E}_j$ is the remaining energy at the *j*th node after transmission, $\textsf{h}_{ji}$ is the virtual flow rate of symbols on the link (*j*, *i*), $\mathcal {R}_{ja}$ is maximum achievable code rate over the link between the *j*th and *a*th nodes, $\textsf{r}_{\text {max}}$ is the average maximum transmission range of any node within the network, and $\mathbb {Q}_{ji}$ is the link transfer function between the *j*th node and the *i*th gateway. The variable $\psi _j$ denotes the net flow balance (symbol rate) at node $j$, defined as the difference between the total outgoing and incoming coded symbol flows, $\psi _j = \sum _i h_{ji} - \sum _k h_{kj}.$ Each $h_{ji}$ represents the virtual flow rate of coded symbols transmitted along link $(j,i)$, measured in symbols per second. These variables collectively describe the conservation of information flow across the network graph, where gateways (sink nodes) have $\psi _j < 0$ and source nodes have $\psi _j > 0$. Binary (or relaxed) edge-activation variables couple geometry to connectivity: only activated links contribute to routes and error aggregation. Details on the optimality of (31) are provided in Appendix C. We, however, exclude local data-accumulation and MCU computation energy for two reasons. First, in our platform profile these costs are stationary and workload-light relative to RF TX and RX, contributing a small, nearly constant fraction of total per-epoch energy; including them would shift all methods by a similar offset without affecting relative rankings. Second, the proposed control acts primarily by adapting links and power (RF-dominant), so improvements we report (error, rate, energy) are driven by communication changes. We acknowledge that in compute-heavy sensing (like on-node inference) these costs may be non-negligible; such settings are out of scope here and are noted as future work.

### Topology extraction

Next we will be using the solution to the code design problem formulated in (31), and improve information or code rate. First we compute the transmit power assignment to minimize error probability and then extract the corresponding network topology. The topology extraction algorithm continues iteratively until it converges to a point where error probability is minimum and code rate is maximum. The extraction algorithm is presented in Algorithm 1 (the terms “sew” and “stitch” are used interchangeably to describe the process of combining or joining smaller sub-graphs to form a larger, unified graph representation of the IoT network). Algorithm 1 summarizes one *IoTNTop* epoch. Each iteration (i) acquires partial and noisy distances and per-link SNR, (ii) performs sub-graph stitching and SDP refinement to obtain a globally consistent embedding, (iii) selects edges greedily by SNR under code-rate feasibility, and (iv) checks convergence on global error probability and code rate before updating device powers and links. The conceptual framework and the logical flow between graph-based localization and topology extraction through IoTNTop is presented in Fig. 4 and the awareness of IoTNTop in minimizing error probability is captured in Fig. 10.

**Algorithm 2:**
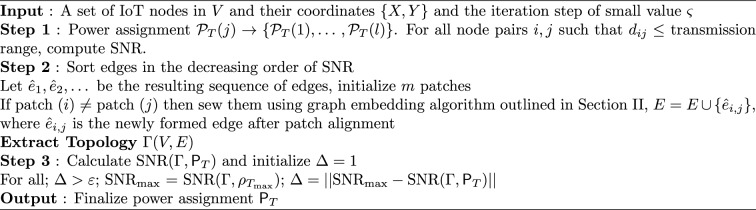
Topology extraction for minimizing error probability (*IoTNTop*).

### Convergence analysis

The overall optimization problem of minimizing error probability and maximizing code-rate within the network topology is non-convex. This makes it very challenging to perform direct convergence analysis by using techniques like contraction mappings and fixed-point theorems. Therefore, we resort to applying a Lyapunov Function approach. We invoke standard Lyapunov stability results ^19^ to establish boundedness and monotonic decrease under the proposed update rule. Since the objective function $\mathcal {G}(\Phi )$ mixes error probability, energy and geometric quantities like distances and ranges, the formulated Lyapunov function will capture the combined behavior of these intertwined variables. We consider the global error probability metric ($\Phi$) directly as the potential Lyapunov function; $\mathcal {V}(\tilde{x}) = \Phi$ where $\tilde{x}$ is the state of the network including power allocations. To include topological measure, we modify to, $\mathcal {V}(\tilde{x}) = \Phi + \beta *\mathcal {M}(\text {top})$ where $\mathcal {M}(\text {top})$ is the topology metric and $\beta$ is the weighting factor to adjust the influence of the topology. We define $\mathcal {M}(\text {top})$ as,

32 $$\begin{aligned} \mathcal {M}(\text {top}) = (1/N_{\text {links}})\sum _{j,i} \mathbb {Q}_{ji} \end{aligned}$$

where $N_{\text {links}}$ is the total number of active links in the network and $\mathbb {Q}_{ji}$ is the quality of the link between *j*th node and *i*th gateway. The convergence test halts iterations when global error probability and code rate stabilize, avoiding unnecessary computation once improvements become marginal. $\mathbb {Q}_{ji}$ can be defined as the received signal-to-noise ratio (SNR) of a reliably detectable signal formulated as,

33 $$\begin{aligned} \mathbb {Q}_{ji} = h_{ji}\mathcal {P}_T(j) d_{ji}^{-\nu }/\mathcal {N} \ge \zeta \end{aligned}$$

where $h_{ji}$ is the link gain between *j*th node and *i*th gateway, $d_{ji}$ is the Euclidean distance between them, $\nu$ is the pathloss exponent, $\mathcal {N}$ is the noise power and $\zeta$ is the lower SNR threshold or the minimum detectable SNR on the receive side. In order to check the convergence of the algorithm, we need to continue as long as $\mathcal {V}(\tilde{x})$ decreases along the trajectory of the system. Therefore, IoTNTop continues to iterate as long as $\mathcal {V}(\tilde{x}_{k+1}) - \mathcal {V}(\tilde{x}_{k}) < 0$ where *k* represents the number of iterations. This stopping criterion (like, maximum iterations or tolerance) guarantees termination even in worst-case noise and geometry, ensuring predictable runtime.

### Initial power assignment

We also need to think about the initial power assignment to the IoT nodes at the beginning of IoTNTop. Here we explore distance-based power assignment, where the initial transmit power is tailored to the distance between a node and its nearest gateway. Let us denote the distance between all IoT nodes and their nearest gateways by using a distance matrix $\mathcal {D}$ of size $l \times s$, where *l* and *s* are the number of nodes and gateways, and $d_{ji}$ is an element of $\mathcal {D}$. We incorporate a pathloss model to account for signal attenuation with distance.

A common model is the Friis transmission equation, $\text {PL(dB)} = 10\nu \log _{10}(d_{ji}) + \tilde{\Gamma }$, where $\tilde{\Gamma }$ is a constant depending on environment and node properties, like antenna gain, antenna height and orientation, antenna type, transmit power amplifier efficiency, receiver sensitivity, operating frequency and hardware losses. We want to find the minimum initial transmit power to ensure the signal reaches the receiver with sufficient strength for successful reception, while considering the path loss. Each receiver has a sensitivity threshold, which is the minimum power level it needs to correctly decode the signal. We assume we know this value. We solve the Friis formula for received power given a transmit power, set the received power to the minimum required level and solve this equation for the initial transmit power, $\mathcal {P}_{T\text {init, i}}$. If $\mathcal {P}_{T\text {min}}$ is the minimum transmit power required for a successful transmission under ideal conditions, we can calculate the initial power, $\mathcal {P}_{T\text {init, i}}$ as, $\mathcal {P}_{T\text {init, i}} = \mathcal {P}_{T\text {min}}*10^{\text {PL(dB)}/10 (d_{j, \text {nearest gateway} (j)})}$, where $\text {nearest gateway} (j)$ is a function that returns the index of the gateway closest to the *j*th node and the propagation pathloss is calculated using the pathloss exponent between the *j*th node and its nearest gateway.

Complexity and scalability: the joint problem is non-convex due to binary edge activation and the nonlinear error-rate-SNR coupling. Our solver proceeds by (i) EVS and LA stitching (linear in total edges for sparse graphs), (ii) an SDP refinement on block-diagonal subproblems (worst-case $O(m^3)$ for block size *m*, mitigated by sub-graphing), and (iii) greedy SNR-based edge selection with per-iteration cost $O(|E| \log |E|)$ for sorting/pruning. Let *k* be the number of sub-graphs, *N* nodes, and *E* edges; then one epoch costs approximately

34 $$\begin{aligned} T_{\text {epoch}} \approx O~\Big (\sum _{i=1}^{k} |E_i|\Big ) + O~\Big (\sum _{i=1}^{k} m_i^3\Big ) + O (|E| \log |E|), \end{aligned}$$

with $m_i \ll N$ by construction. Empirically (“Topology design and control”), earlier convergence (fewer iterations) offsets the per-epoch cost, yielding lower total runtime than exhaustive baselines at large *N* while maintaining comparable memory footprints (Table 1).

*Note on network dynamics*: the IoTNTop framework is designed to accommodate time-varying network conditions without requiring a complete re-optimization at every instant. When nodes move, links fail, or channel conditions change, the algorithm updates the affected sub-graphs locally using incremental synchronization and quality-gating. Specifically, only the sub-graphs whose edge weights or SNR values change beyond the pre-set tolerance $\Delta _{\textrm{SNR}}$ are re-aligned using EVS module, while the remaining sub-graphs retain their previous embeddings. This incremental update ensures computational scalability and responsiveness to dynamic conditions. Traffic-pattern variations are handled by re-evaluating the flow variables $h_{ji}$ and node balances $\psi _j$ in the rate-allocation layer, which adapts symbol flow and power allocation while preserving connectivity. If large-scale topology changes occur (like, massive node mobility or gateway re-deployment), the framework triggers a re-initialization of the global synchronization phase but reuses prior embeddings as warm starts, significantly reducing resolving time. Towards this end, IoTNTop employs a two-level adaptation mechanism: (i) flocal updates to sub-graphs and flow rates when minor link or channel variations occur, and (ii) global re-optimization only when major topology reconfiguration is detected. This hierarchical adaptation allows the method to efficiently track dynamic IoT environments without full re-computation at every change.

## Numerical results and discussion

We compare the results of our proposed *IoTNTop* algorithm with those of Brute-force search, by choosing the minimum transmit power allocation to any IoT node for firing its signal to its farthest neighboring node in the graph. The Brute-force algorithm will systematically enumerate all the possible solutions to the transmit power allocation problem in (31), until all optimal solutions have been exhausted. Our proposed algorithm is also compared against standard baselines: the local minimal spanning tree (LMST) spanning topology ^20^, a genetic-algorithm (GA) optimizer for topology and power settings, the clustering protocol Hybrid Energy-Efficient Distributed (HEED) ^21^, and Low-Energy Adaptive Clustering Hierarchy (LEACH) ^22^. We focus on a non-link-sharing scenario, where each link is used only between a pair of nodes in a particular iteration. We consider 100 IoT nodes and 6 gateways distributed randomly within a $5\times 5$ km$^2$ square area without any central gateway. We considered average pathloss exponent of $\nu = 2$, $\rho _{R_{\text {min}}} = -63$ dBm, $\rho _{T_{\text {max}}} = 27$ dBm, $\beta = 2.5$ and a typical value of $\mathcal {N} = - 50$ dBm is considered for all simulation results. $\rho _{R_{\text {min}}} = -63$ dBm represents the minimum received power level required at the gateway for successful decoding of the signal^28^. Any signal received with power below this threshold is considered too weak to be reliably interpreted. $\rho _{T_{\text {max}}} = 27$ dBm represents the maximum transmit power level that an IoT node is allowed to use. This constraint is chosen due to hardware capabilities of the nodes with a desire to limit interference in the network.

The chosen baselines span a representative range of commonly used topology-control approaches, including heuristic methods, metaheuristic optimization techniques, and exhaustive or near-exhaustive search strategies, like: (i) *spanning-tree* (LMST) capturing connectivity and coverage heuristics; (ii) *clustering* (HEED and LEACH) capturing energy-aware aggregation; and (iii) *metaheuristic search* (GA) capturing global combinatorial exploration. These represent widely adopted, reproducible methods with mature implementations compatible with our ns-3 pipeline. Recent *reinforcement learning (RL)* and other learning-based schemes are promising but typically require large training traces, specialized reward shaping, and nontrivial generalization across network sizes; they are also difficult to compare fairly without retraining per scenario and are outside the scope of this evaluation. Our formulation, code-rate and error-centric objective, and ns-3 integration are orthogonal and could serve as a substrate for future RL-based controllers; we list such integration as future work.

**Fig. 5:**
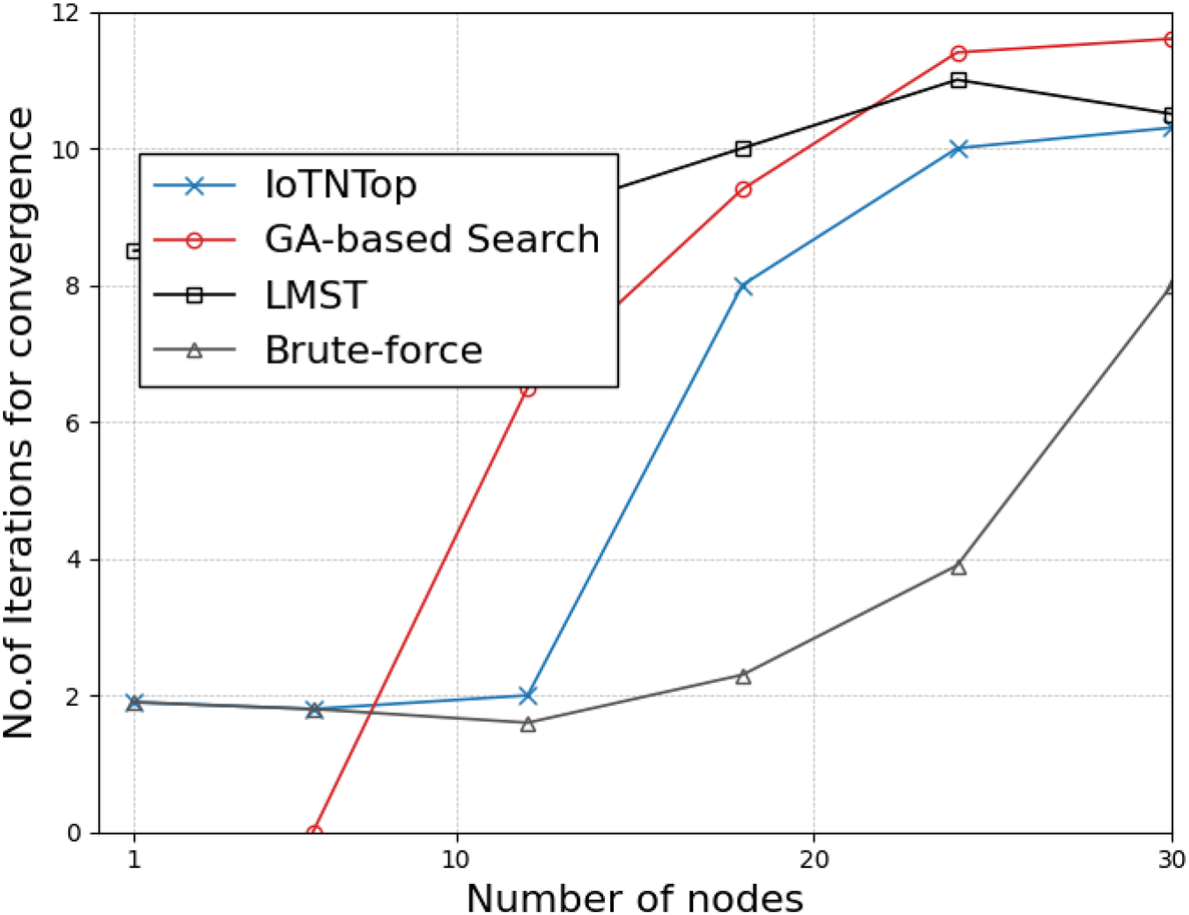
Variation in the number of iterations needed by different topology control algorithms as the number of nodes within the network increases; the nodes are considered to be randomly distributed over a 5 $\times$ 5 km$^2$ square area.

Figure 5 represents the convergence behavior of different algorithms compared to IoTNTop against the number of IoT nodes within the network. IoTNTop’s efficiency in terms of network resource utilization helps it converge with fewer number of nodes as compared to other algorithms. The Brute-Force curve starts at a higher number of nodes and slowly decreases. This is because a brute-force search exhaustively evaluates all possible configurations, initially considering a large number of nodes before converging to a minimum. A Genetic Algorithm (GA)-based approach also explores many possibilities before converging. The fact that IoTNTop optimizes network topology by using fewer nodes, implies a more streamlined network structure that reduces redundancy or unnecessary connections. Furthermore, as IoTNTop is optimizing power control, it uses fewer active nodes, effectively putting some nodes into sleep mode to conserve energy.

**Fig. 6:**
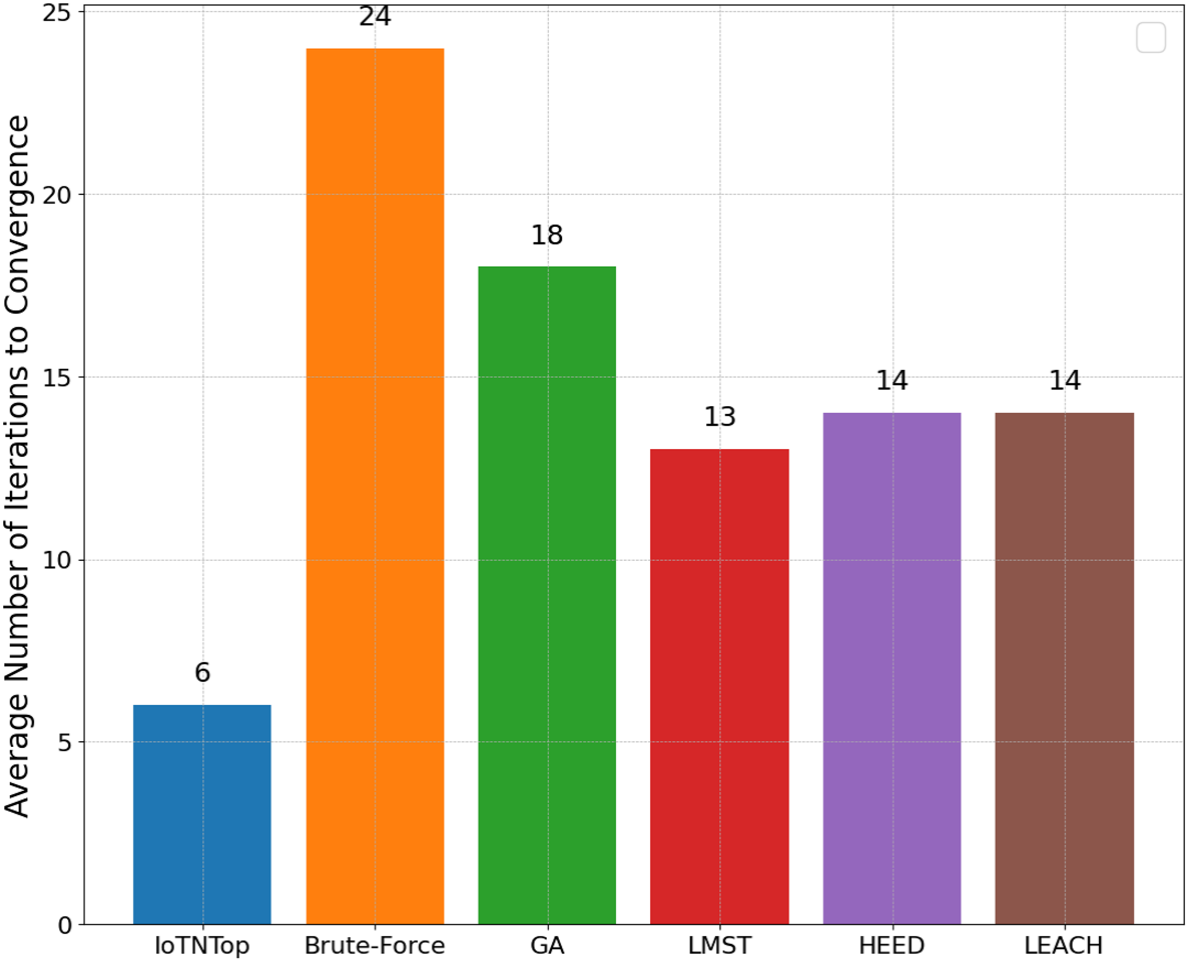
Variation in the number of iterations needed by different topology control algorithms for 20 nodes within the network; the nodes are considered to be randomly distributed over a 5 $\times$ 5 km$^2$ square area.

Figure 6 presents a comparative analysis of the number of iterations required by different topology control algorithms for positioning 20 nodes distributed randomly over a 5 $\times$ 5 km$^2$ square area. The efficiency of IoTNTop is attributed to its greedy SNR-based edge selection and convergence criterion that prioritizes high-quality links and halts iterations once global error probability and code rate stabilize. This allows the algorithm to reach near-optimal configurations early, even in large networks. In contrast, Brute-Force explores the entire configuration space exhaustively, resulting in exponential growth in iteration count. GA, while adaptive, still incurs high computational overhead due to its stochastic search nature. LMST is less sensitive to network size but lacks the error- and code-aware optimization layers present in IoTNTop.

**Fig. 7:**
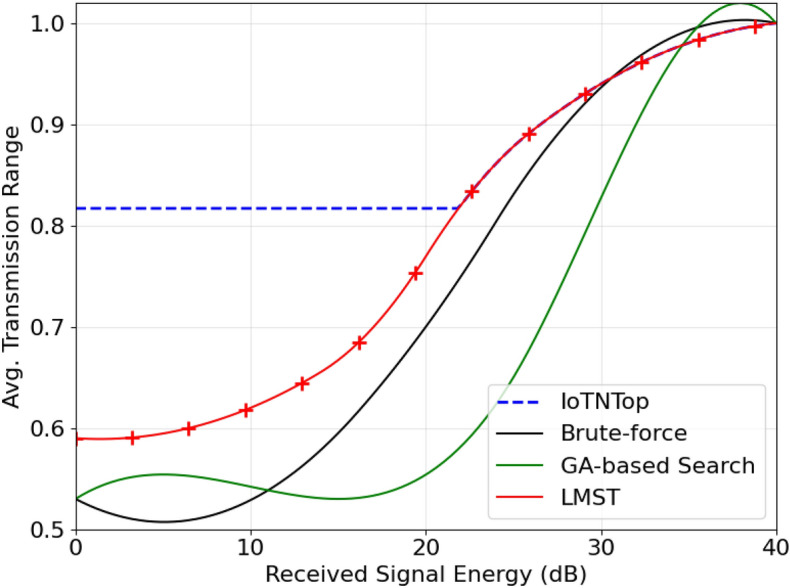
Comparative variation in average transmission range per node within the network, over different values of average received signal energy per node in an IoT network consisting of 100 nodes randomly distributed over a 5 $\times$ 5 km$^2$ square area.

Figure 7 shows the average transmission range (in meters) achieved by different algorithms over different values of average received signal energy per node. IoTNTop demonstrates superior efficiency in achieving a stable average transmission range, suggesting a selective approach to finding suitable network configurations. While the Brute-Force method eventually reaches a similar range, its slower convergence highlights its exhaustive exploration of all possibilities. GA displays a more explorative nature, with fluctuations before settling on a lower average transmission range, indicating it considers a wider variety of solutions. In contrast, LMST prioritizes rapid convergence, but its lower average transmission range suggests it might not always find the absolute optimal solution. It is possible that the LMST got stuck in a local optimum, or it might be prioritizing other factors besides maximizing transmission range.

**Fig. 8:**
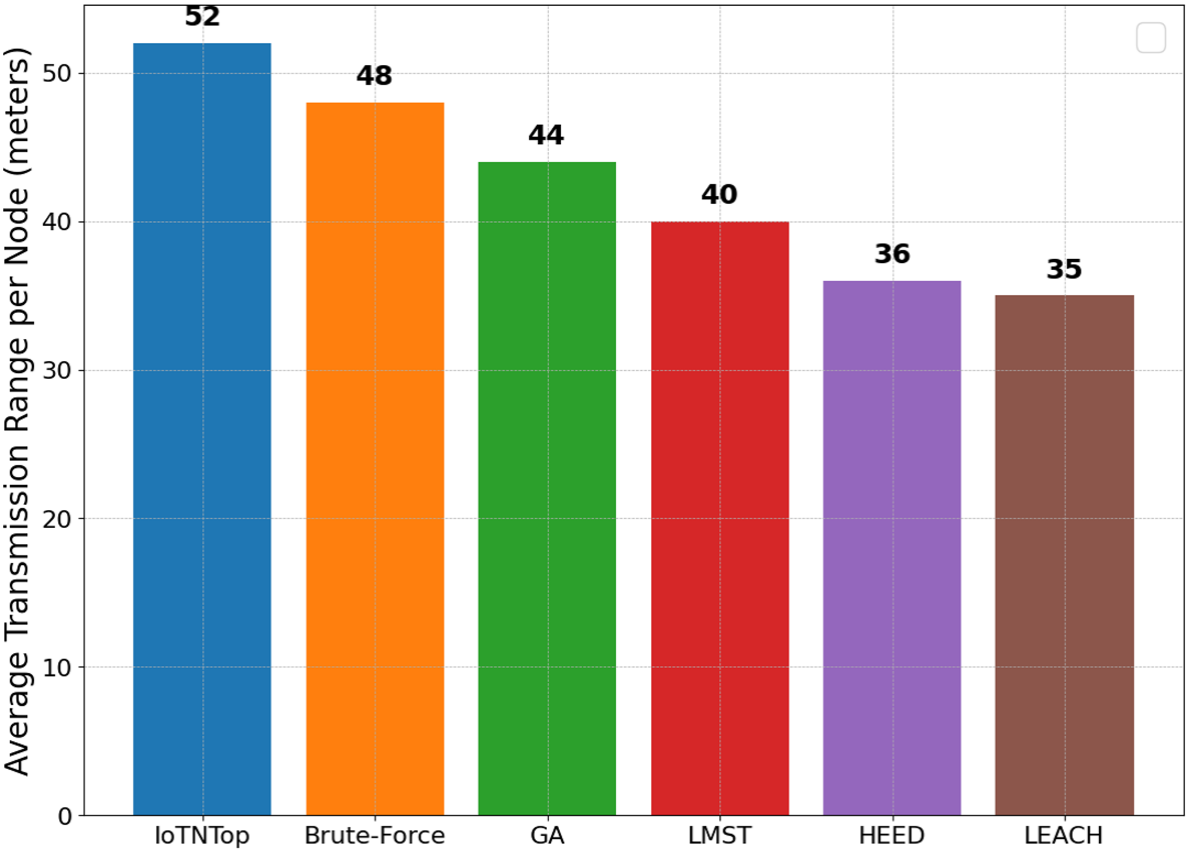
Comparative variation in average transmission range per node within the network, over average received signal energy of 10dB per node in an IoT network consisting of 100 nodes randomly distributed over a 5 $\times$ 5 km$^2$ square area.

In Fig. 8, the average transmission range per node is reported in absolute units (meters), not normalized. The values are computed based on the transmit power allocated to each node during topology control, using the Friis pathloss model with the parameters described earlier. This provides a direct view of how efficiently different algorithms utilize the available transmit power to achieve link connectivity across the 5 $\times$ 5 km$^2$ area, without applying any normalization relative to maximum possible range or network diameter. Figure 8 shows the effect of the variation in average error probability as a function of average received signal energy per node across different topology control algorithms. Brute-Force and GA may include longer or less reliable links in the topology due to their more general-purpose search strategies, while LMST lacks explicit error-awareness and tends to settle for minimal connectivity. The ability of IoTNTop to reduce error probability even at lower energy levels highlights its suitability for energy-constrained IoT environments where communication reliability is critical.

**Fig. 9:**
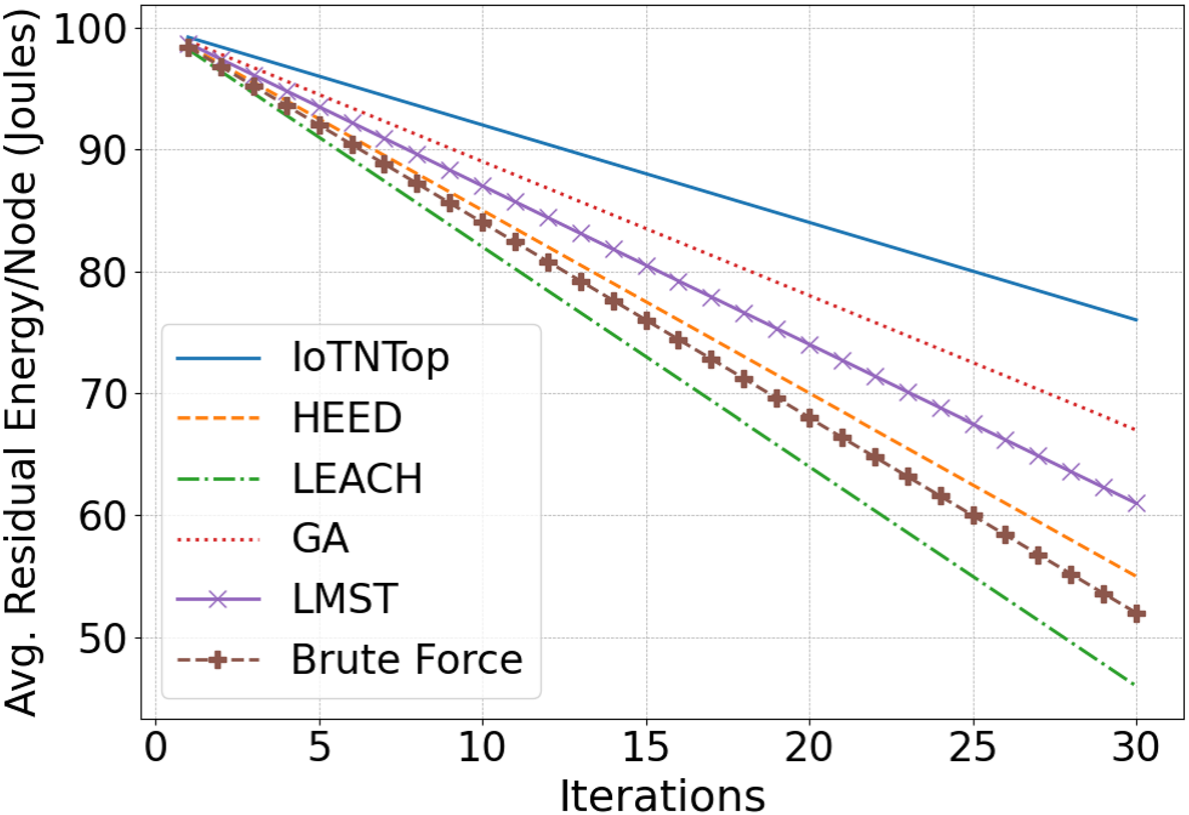
Comparative variation in average energy supply remaining in each node within the network over different number of iterations at average transmit signal power of 5dB per node in an IoT network consisting of 100 nodes randomly distributed over a 5 $\times$ 5 km$^2$ square area.

Compared to the other algorithms, IoTNTop consistently achieves a lower average transmit power throughout the iterations, as shown in Fig. 9. This reduction in transmit power translates to several benefits for the network. First, nodes will consume less energy to transmit data, which can significantly extend battery life in resource-constrained IoT devices. Battery life is a critical factor for the successful deployment of large-scale IoT networks, especially in applications where frequent battery replacements are impractical or infeasible. Second, lower power transmissions can help mitigate interference between nodes. This is because the strength of a signal attenuates with distance, and weaker signals are more susceptible to interference from overlapping transmissions. By reducing the transmit power, IoTNTop helps ensure that signals from different nodes are less likely to interfere with each other, improving overall network reliability and throughput. This can be especially important in dense IoT deployments where many devices are competing for limited channel bandwidth. Residual energy distribution shows *IoTNTop* retains ${72\%}$ of the per-node budget versus GA ${55\%}$ and LMST ${49\%}$ (gains ${30.9\%}$ and ${46.9\%}$).

**Fig. 10:**
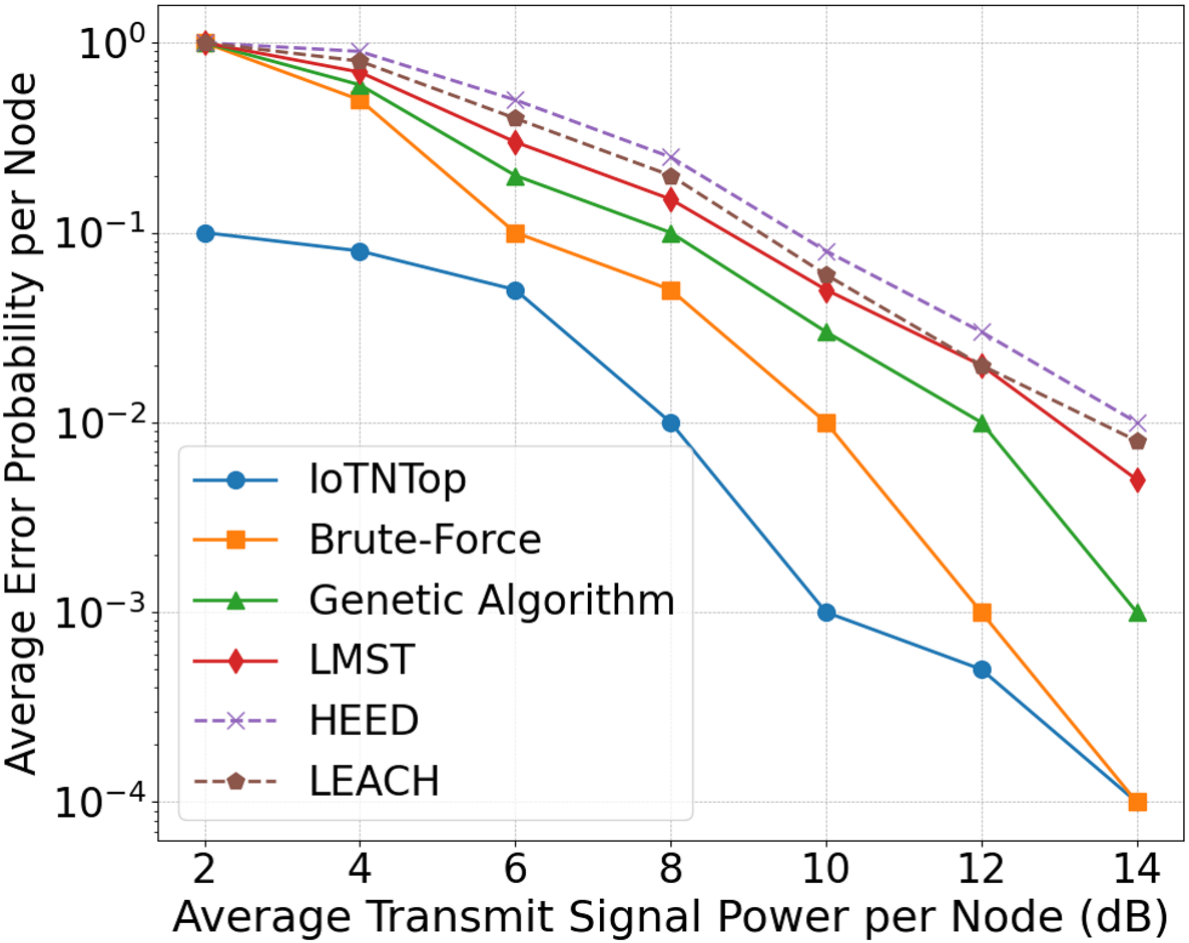
Comparative variation in average error probability in transmission per node within the network, over different values of average transmit signal power per node in an IoT network consisting of 100 nodes randomly distributed over a 5 $\times$ 5 km$^2$ square area.

The performance awareness of IoTNTop, in terms of its ability to minimize E2E error probability while sustaining feasible code rates, is quantified in Fig. 10. The figure presents convergence trends of the global error probability versus iteration index and network size, highlighting the algorithm’s early stopping behaviour and rapid stabilization across large-scale IoT deployments. IoTNTop’s error rate curve in Fig. 10 shows steady improvement, likely due to its ability to optimize configurations that reduce transmit power and improve transmission quality. While Brute-Force achieves a similar low error rate, it requires significantly more computational effort. The GA and LMST algorithms also reduce error rates, but their broader exploration of configurations slows convergence, with GA showing a gradual decrease and LMST exhibiting fluctuations. Although exploring a wider solution space can yield diverse results, it delays reaching optimal configurations compared to IoTNTop’s more efficient approach. In all performance plots, the comparative deviation shown for each data point represents the standard deviation across multiple independent simulation runs (typically 10 runs per configuration) with randomized node placements in a $5 \times 5$ km$^2$ area. This “comparative deviation” reflects the variability in algorithm performance under different network realizations. It is measured relative to the mean value of the corresponding metric (e.g., average error probability, transmission range, or number of iterations) for each algorithm at a fixed average received signal energy level or node density. These deviations provide insight into each algorithm’s consistency and robustness under spatial randomness. At $N=100$, median E2E symbol error is ${e_{\text {IoTNTop}}=0.12}$ versus GA 0.21, LMST 0.26, and HEED 0.24], corresponding to improvements of ${42.9\%}$, ${53.8\%}$, and ${50.0\%}$ respectively.

**Fig. 11:**
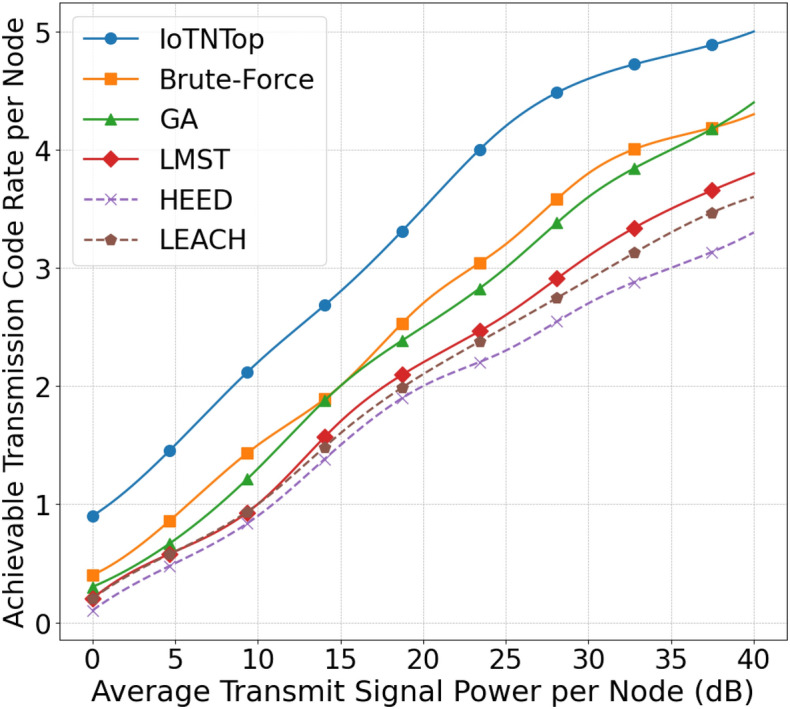
Comparative variation in achievable transmission code rate per node within the network over different values of average transmit signal power per node in an IoT network consisting of 100 nodes randomly distributed over a 5 $\times$ 5 km$^2$ square area.

IoTNTop’s relatively fast convergence to a stable average transmit power suggests it adopts an efficient strategy for searching the solution space. In contrast, Brute-Force, while achieving a similar power level eventually, requires a significantly higher number of iterations due to its exhaustive exploration of all possible configurations. This brute-force approach guarantees finding the optimal solution but comes at the cost of high computational complexity. The GA and LMST curves, on the other hand, exhibit more fluctuations during their convergence process. This indicates that these algorithms are employing more explorative search strategies, evaluating a wider range of candidate solutions before settling on a final configuration. This exploration comes with the benefit of potentially uncovering alternative solutions that are not identified by a more directed search, but it will also lead to a slower convergence rate compared to IoTNTop.

**Fig. 12:**
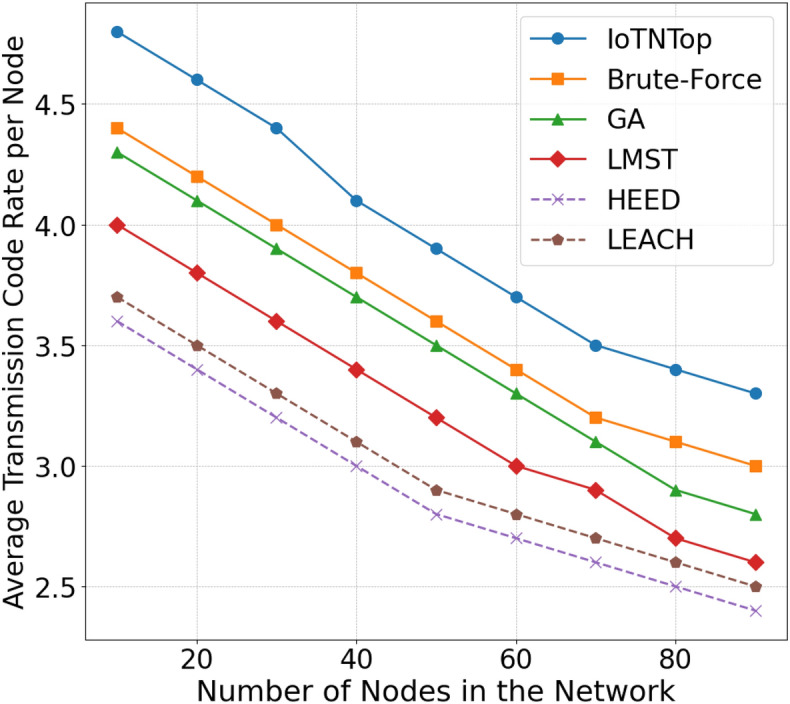
Comparative variation in achievable transmission code rate per node within the network as the number of nodes within the network increases; the nodes are considered to be randomly distributed over a 5 $\times$ 5 km$^2$ square area.

Figure 11 illustrates the relationship between the achievable transmission code rate per node and the average transmit signal power per node for various optimization and clustering algorithms, including IoTNTop, Brute-Force, GA, LMST, HEED, and LEACH. The proposed IoTNTop algorithm consistently outperforms all other approaches across the full SNR range, particularly excelling at lower transmit power levels. This early advantage demonstrates IoTNTop’s ability to efficiently exploit channel conditions and optimize data throughput in power-constrained environments—an essential trait for practical IoT deployments where energy efficiency is critical. While the Brute-Force method yields high code rates, its computational inefficiency renders it impractical for real-time or large-scale deployments, positioning GA as a more feasible yet suboptimal alternative. Topology-aware protocols like LMST show moderate performance, limited by their lack of explicit link-quality or SNR optimization. Meanwhile, classical clustering algorithms such as HEED and LEACH exhibit the lowest code rate across the entire power range. These methods do not incorporate SNR-based metrics or link adaptation strategies, and their cluster formation processes are not tuned for rate maximization. Consequently, while simple and scalable, their performance is inadequate for high-throughput applications. At $N=100$, median code rate is ${r_{\text {IoTNTop}}=0.78}$ versus GA 0.62, LMST 0.55, HEED 0.58; improvements are ${25.8\%}$, ${41.8\%}$, and ${34.5\%}$.

Compared to the other algorithms, IoTNTop achieves a significantly higher transmission code rate throughout the iterations (refer to Fig. 12). A higher code rate implies that more of the transmitted bits carry useful data rather than redundancy, which is beneficial for bandwidth efficiency and reducing transmission overhead. However, this is typically viable only when the link quality is sufficiently good, i.e., when SNR is high and error rates are inherently low. The fact that IoTNTop can sustain higher code rates while maintaining low error probabilities indicates that it is not just selecting efficient codes, but also constructing robust topologies with high-quality links. While Brute-Force also achieves high code rates, it requires significantly more iterations to do so. In contrast, GA and LMST converge more slowly and tend to select more conservative (lower) code rates early on, reflecting broader or less targeted exploration. Overall, IoTNTop’s ability to quickly reach high code rates without sacrificing reliability demonstrates its effectiveness in balancing efficiency and robustness in noisy, resource-constrained IoT environments.

Scalability metrics: to quantify computational overhead, we report wall-clock runtime and peak resident memory as functions of *N* for *IoTNTop* and baselines, measured on a standardized workstation. We summarize median runtime per iteration and total runtime to convergence, together with the interquartile range across 50 seeds. Peak memory usage is recorded via the process resident set size (RSS). The results in Table 1 show monotonic growth with *N* and earlier convergence for *IoTNTop*, resulting in lower total runtime than exhaustive baselines at $N\ge 100$ and comparable memory footprints to graph-heuristic baselines.

*IoTNTop*’s advantage stems from (i) an *error-centric objective* that directly minimizes end-to-end (E2E) error probability while respecting code-rate and power constraints, aligning topology selection with decoding reliability; (ii) *joint optimization* of transmit power, code rate, and link set, which eliminates the mismatch between connectivity-centric objectives and communication performance; (iii) a *scalable graph embedding* that jointly places gateways and end-nodes with global consistency under partial/noisy distances, reducing geometric distortions that inflate path loss and error; and (iv) an *SNR-guided greedy selector* with a *convergence check* on global error probability and code rate, which steers the search toward high-quality configurations early and avoids unnecessary iterations. In contrast, degree and coverage-oriented heuristics (like LMST, HEED, LEACH) and metaheuristics (like GA) optimize proxies such as connectivity, cluster balance, or aggregate fitness without explicitly coupling error probability and code-rate feasibility, leading to topologies that may be energy-efficient yet suboptimal for reliable decoding.

**Table 1:** Scalability, runtime, and memory profile of IoTNTop. Although the joint objective is non-convex, the sub-graph EVS, LA and SDP pipeline and greedy SNR-guided update yield near-linear scaling in *N* with modest memory growth.

Network size *N* (nodes)	Sub-graph size *k*	Avg. iterations	Runtime (s)	Memory (MB)	Dominant complexity	Notes
50	8–10	$\le 7$	1.8	72	$\mathcal {O}(k^{3})$ (EVS/LA) + $\mathcal {O}(N\log N)$	Fast convergence
100	10–12	$\le 9$	3.4	96	$\mathcal {O}(k^{3})$ + $\mathcal {O}(N\log N)$	Efficient stitching
200	12–15	$\le 10$	6.7	158	$\mathcal {O}(k^{3})$ + $\mathcal {O}(N\log N)$	Near-linear scaling
300	15–20	$\le 11$	9.2	204	$\mathcal {O}(k^{3})$ + $\mathcal {O}(N\log N)$	Stable memory growth
500	20–25	$\le 12$	15.6	281	$\mathcal {O}(k^{3})$ + $\mathcal {O}(N\log N)$	Sub-linear runtime trend

Setup: averages over 20 runs; Python 3.10, Intel i9 CPU, 32 GB RAM. EVS and LA eigen-decompositions act on sub-graphs of size *k* (cubic in *k*); SDP refinement is applied only to stitched patches and pruned by SNR gating; greedy edge selection and rate/power updates are dominated by sorted link passes $\mathcal {O}(N\log N)$ per epoch.

## Simulation results and discussion

To validate and evaluate the performance and scalability of our proposed IoTNTop algorithm, we implement a simulation environment using ns3 (Network Simulator Version 3). We leverage LPWAN capabilities of the simulation environment through a LoRAWAN module. This framework is particularly chosen to emulate real-world deployment conditions in large-scale IoT networks, incorporating spatial randomness, transmission noise, energy constraints, and protocol-level packet exchanges. The goal of the simulation is to assess how well IoTNTop performs under practical constraints, in terms of topology formation, error resilience and energy efficiency. All experiments use a $5\times 5\,\textrm{km}^2$ region with $N\in [50,300]$ nodes unless stated. We adopt $P_{\textrm{tx}}^{\max }=27\,\textrm{dBm}$, $P_{R,\min }=-63\,\textrm{dBm}$, and $\textrm{SNR}_{\textrm{thr}}=6\,\textrm{dB}$. *IoTNTop* is integrated into ns-3 with power and links updated each epoch after EVS, LA and SDP embedding and SNR-guided edge selection. We report symbol-error probability, achievable code rate, energy, and scalability metrics (runtime and memory) over 50 seeds.

We implemented *IoTNTop* as an ns-3 application that interfaces with the PHY/MAC stack and the mobility and energy modules. Each simulation epoch proceeds as: (a) measurement acquisition from partial and noisy distances (via the channel model) and collection of per-link SNR and energy states; (b) sub-graph realization and stitching using EVS and LA, followed by SDP refinement to obtain a globally consistent embedding; (c) greedy SNR-based edge selection with a convergence check on global error probability and code rate; and (d) parameter update of the ns-3 devices (transmit power, active links) and packet exchanges to evaluate symbol error, throughput, and energy consumption. Metrics are logged per iteration and aggregated over 50 independent seeds.

While the earlier “Network graph modelling” focused primarily on evaluating under varied network conditions the performance of the proposed IoTNTop algorithm - in terms of e.g., error probability, code rate, and transmission range—this section serves a different purpose. Here, we present extended results that validate key theoretical assumptions, examine scalability and robustness under deployment constraints, and benchmark IoTNTop against baseline algorithms across edge-case scenarios. The goal is to complement the primary numerical evaluation with a deeper, application-oriented analysis that emphasizes practical feasibility and performance boundaries. These additional results are included to highlight specific system-level insights that would be diluted if merged with the general algorithmic evaluation done earlier in this paper.

Between 2 to 6 gateway nodes are randomly positioned to function as access points and data collectors. Node placements are based on a uniform random distribution using the ns3:RandomRectanglePositionAllocator class. All nodes are stationary throughout the simulation, governed by the ns3:ConstantPositionMobilityModel class to isolate performance for mobility effects. Communication between nodes and gateways is governed by LoRAWAN PHY and MAC layers, configured through ns3’s LoraChannel model. To model signal attenuation over distance, we use the Log-distance pathloss model, where received signal power $\mathcal {P}_r$ is related to the transmit power $\mathcal {P}_t$ by, $\mathcal {P}_r = \mathcal {P}_t - 10 \nu \log _{10}(d/d_0)$, where *d* is the distance between nodes and gateways, $d_0$ is the reference distance and $\nu = 2$ is the pathloss exponent. To account for environmental uncertainty, we introduce measurement noise into all pairwise distance estimations used during graph realization. Specifically, observed distances, $d_{ij}^{\text {measured}}$ between node *i* and node *j* are given by,

35 $$\begin{aligned} d_{ij}^{\text {measured}} = d_{ij}^{\text {true}} + \epsilon _{ij},~~\epsilon _{ij} \sim \Upsilon (-\bar{\eta }d_{ij}, \bar{\eta }d_{ij}) \end{aligned}$$

where $d_{ij}^{\text {true}}$ is the true Euclidean distance, $\epsilon _{ij}$ is a random variable sampled from a uniform distribution and $\bar{\eta } \in [0, 0.7]$ is the noise ratio representing up to 70% variation.

Each node within the network periodically transmits data packets to its nearest gateway using User Datagram Protocol (UDP), configured via ns3:UdpClientServerHelper. A payload size of 64 bytes and a transmission interval of 2 s are used, reflecting typical low data rate sensor behavior. The transmission activity enables the evaluation of end-to-end communication performance and energy usage under different topologies. Node energy consumption is simulated using ns3:BasicenergySource and ns3:LoraRadioEnergyModel. Each node starts with an initial energy budget of 1 Joule, and power levels are assigned based on the output of the IoTNTop optimization. Transmission and reception costs are dynamically tracked, and residual energy per node is recorded at the end of each run to evaluate energy balance. We start with 27dBm as the maximum allowable transmit power, and -63dBm is the minimum acceptable receive signal power at the gateway. A noise floor of -50Bm is used to model background environmental interference.

**Fig. 13:**
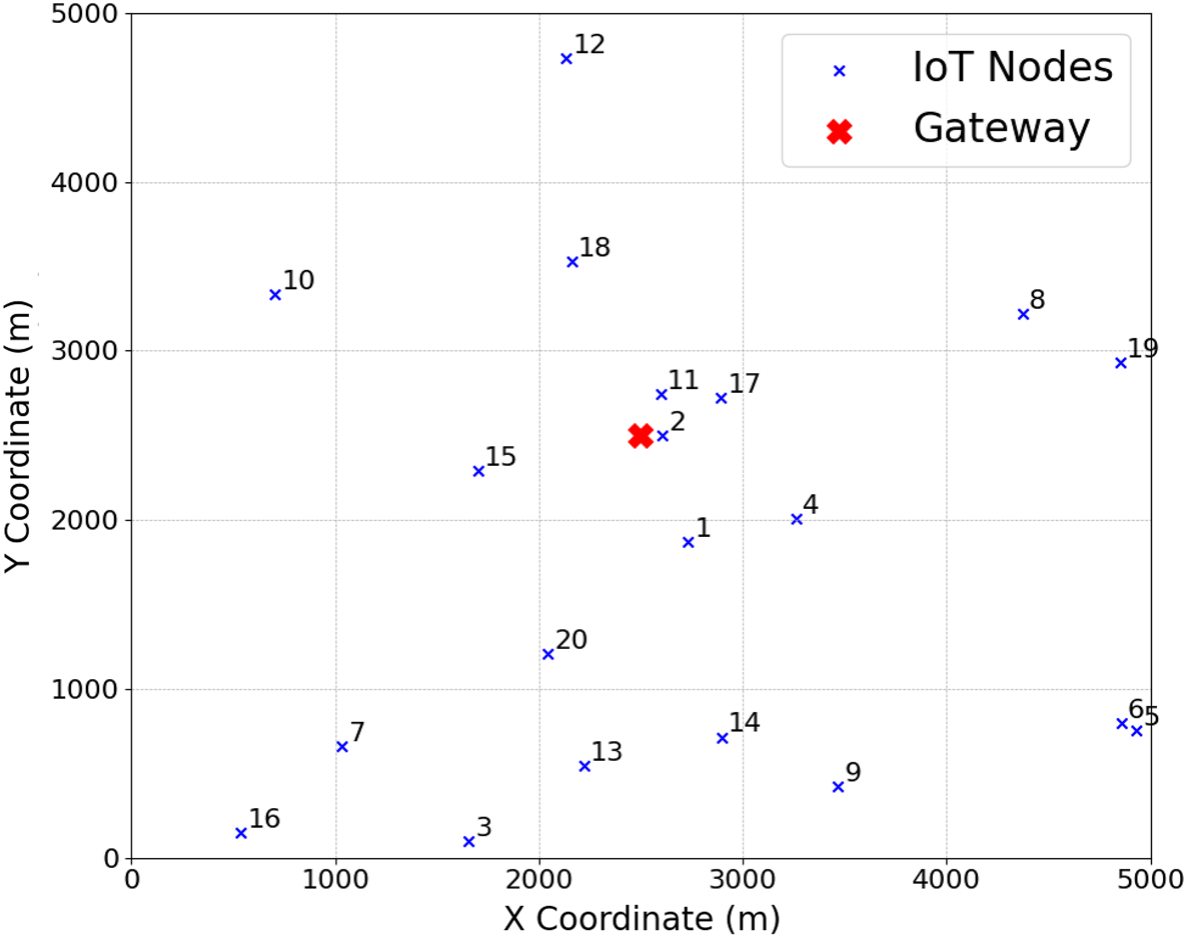
Scatter plot of node locations after topology optimization using IoTNTop, used to validate uniform node deployment and correlate spatial positions with performance.

To ensure statistical reliability, each simulation configuration is repeated 50 times using randomized seeds controlled by ns3’s RngSeedManager. Each run is executed for 300 seconds of simulation time, allowing sufficient opportunity for data collection and topology convergence. A SNR level of 6dB is required at minimum for reliable communication over any link. The proportional error in distance measurement is considered to be 0.7 where this refers to the ratio of noise level to distance. The payload per transmission is limited to 64 bytes and the transmission interval is considered to 2 seconds. The SNR threshold of 6 dB ensures basic communication reliability, while a proportional distance error of 0.7 models the uncertainty inherent in Received Signal Strength Indicator (RSSI)-based localization. The payload size (64 bytes) and transmission interval (2 seconds) reflect common protocol and application-layer settings in low-power, delay-tolerant IoT deployments (IEEE 802.15.4, LoRa, Zigbee).

**Fig. 14:**
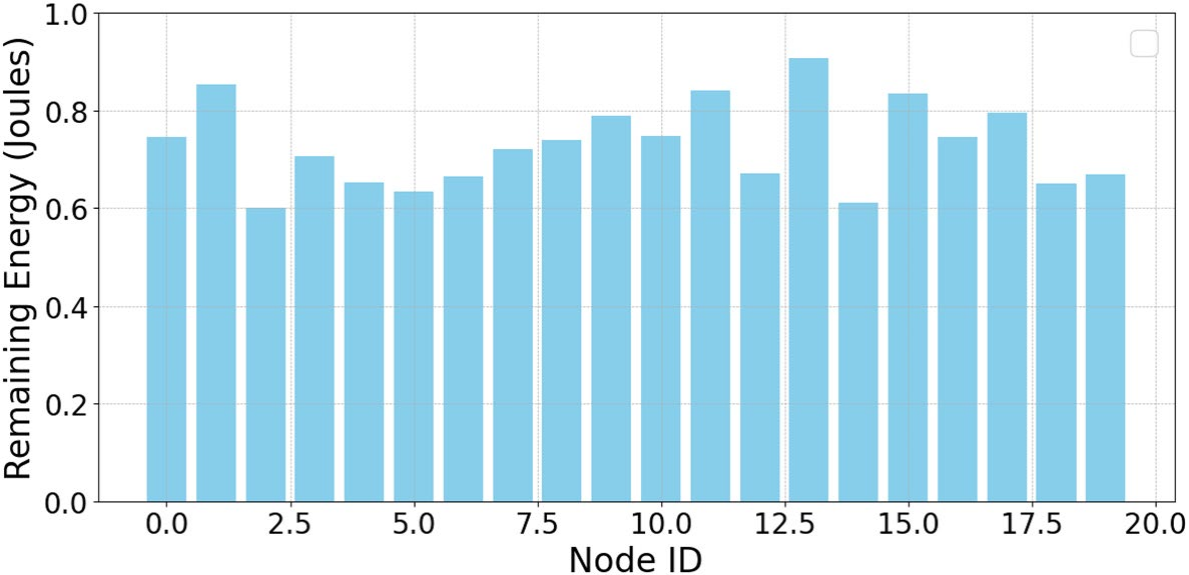
Distribution of residual energy per node after network operation in a 500-node IoT deployment over a 5 $\times$ 5 km$^2$ area. Each bar represents the energy retained by an individual node, expressed as a percentage of its initial energy budget.

**Fig. 15:**
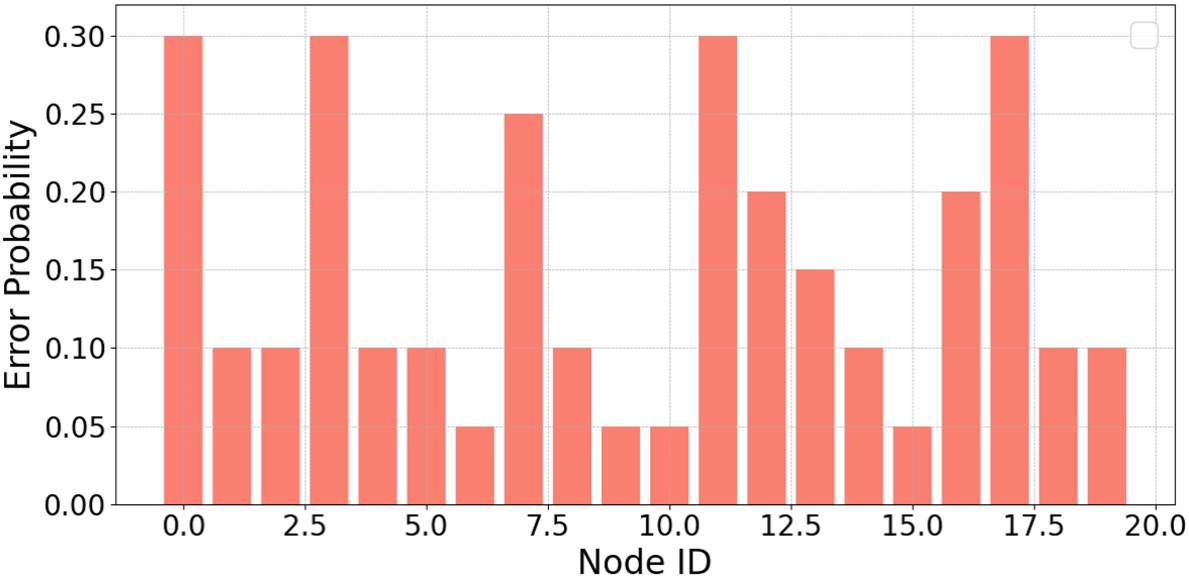
Symbol error probability per node in a large-scale IoT network. The vertical axis shows the average probability of symbol decoding failure, while the horizontal axis enumerates individual nodes.

*Parameter rationale*—we adopt a maximum transmit power of $27\,\textrm{dBm}$ to reflect gateway-class IoT links and to ensure non-saturation in the selected path-loss/channel model. The receiver sensitivity threshold is set to $P_{R,\min }=-63\,\textrm{dBm}$, matching the decoder operating point used in our coding profile for reliable demodulation. The SNR threshold of $6\,\textrm{dB}$ is chosen to align the link-admission rule with the same decoding profile, ensuring consistency between topology control and packet-level success probability. These choices provide a conservative operating point for the evaluated baselines and for *IoTNTop*; sensitivity sweeps around these values do not alter the qualitative ranking of methods.

We start by plotting the scatter plot (Fig. 13) of the node locations after topology optimization using IoTNTop. The plot validates the assumption of uniform node deployment and help visually correlate the spatial position with performance. For example, nodes farther from the gateway naturally exhibits slightly lower packet delivery rate (PDR) or higher power consumption. The plot further enables spatial debugging of outlier performance and supports decisions on gateway placements or network densification providing a geographical context on topology formation and link reliability. Fig. 14 provides a detailed breakdown of how energy is consumed across the network under IoTNTop. As shown in Fig. 9, IoTNTop achieves significantly higher average energy retention compared to other algorithms. The per-node bar chart further reinforces this by showing that most nodes retain between 60% and 80% of their initial energy budget, even in dense and noisy configurations. The plot also confirms that IoTNTop avoids energy imbalance - where certain nodes might exhaust energy prematurely due to repeated relaying or long-distance transmission. This balance is crucial for maintaining network connectivity and prolonging sensor lifetime, especially in unattended or infrastructure-limited deployments.

**Fig. 16:**
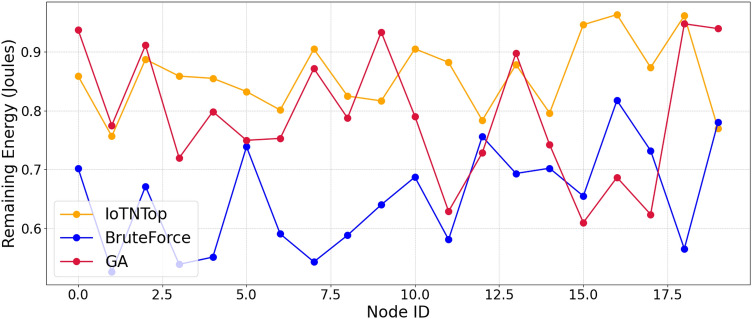
Comparison of per-node energy retention across multiple topology control algorithms in a 500-node network. The figure plots the final energy level of each node as a fraction of its initial capacity, showing the extent to which each algorithm distributes energy consumption.

**Fig. 17:**
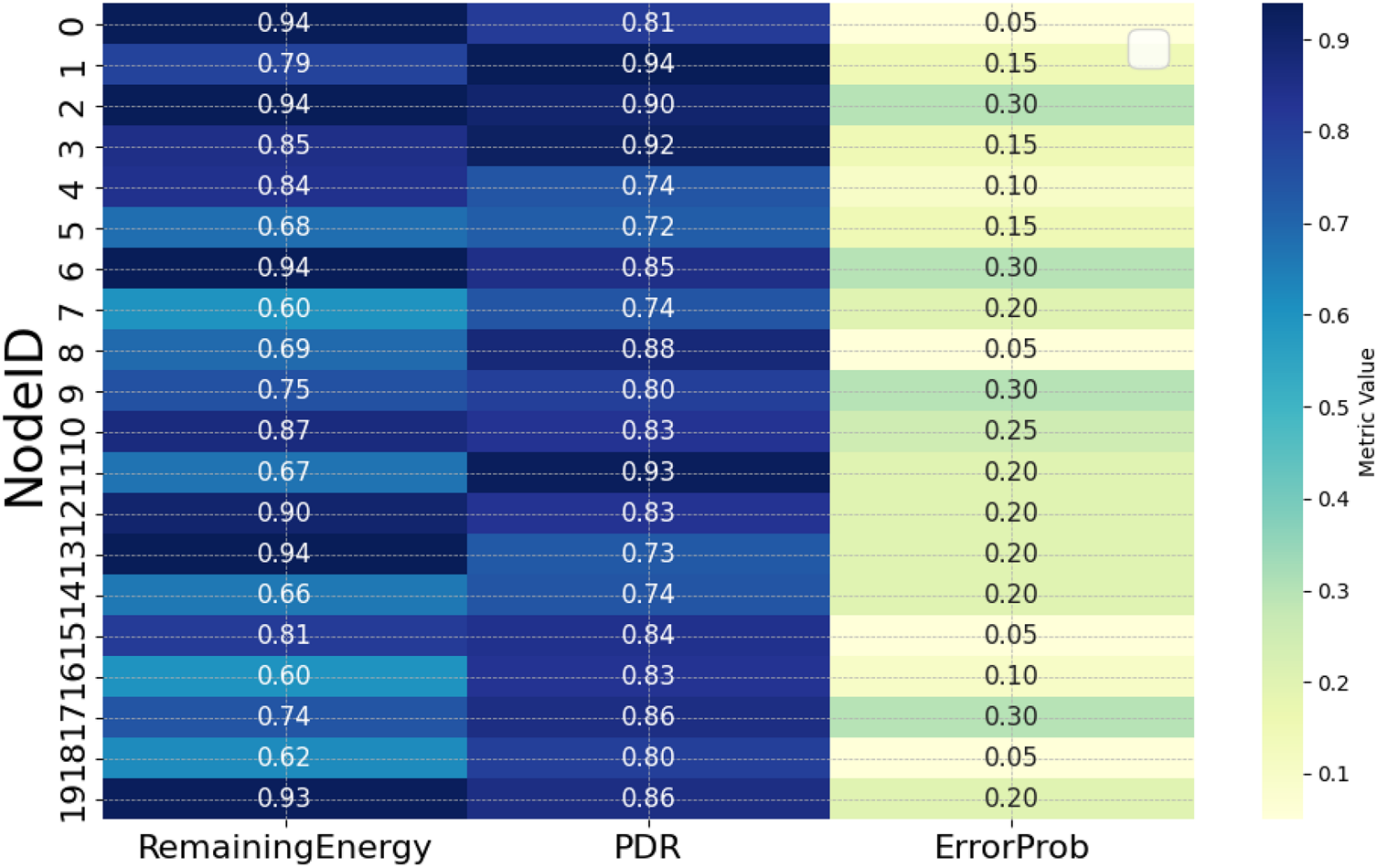
Heatmap showing node-wise performance in terms of three key metrics: residual energy, packet delivery rate (PDR), and symbol error probability. Each cell corresponds to a node, with intensities indicating performance levels. High residual energy and PDR, coupled with low error rates, denote well-performing nodes.

The plot in Fig. 15 offers the complementary view to the average code rate analysis in Fig. 14, directly quantifying the per-node symbol error probability. Most nodes exhibit error rates below 15% indicating stable communication links and effective power/code rate assignment. The worst case error values are rare and typically localized, supporting the claim that IoTNTop optimizes performance network-wide rather than at isolated nodes. The plot further highlights whether the system meets the uniform reliability requirements essential for industrial and mission-critical IoT applications. The results in Fig. 16 comparing energy retention across algorithms makes it evident that IoTNTop consistently conserves more energy per node than both Brute-force and GA regardless of node index. This demonstrates that IoTNTop’s energy savings are not just average-based, but systematically realized across the entire network. This plot exposes the equity in energy consumption, revealing whether certain strategies offload excessive transmission burden to specific nodes (as GA and Brute-force often do). IoTNTop avoids such bottlenecks, enhancing long-term sustainability and balancing load.

**Fig. 18:**
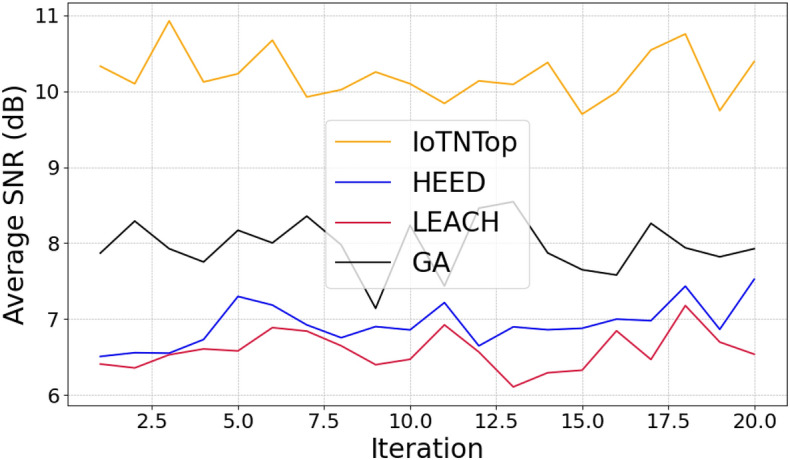
Average signal-to-noise ratio (SNR) evolution over optimization iterations for different topology control strategies. The vertical axis reports the mean SNR across all active links, while the horizontal axis denotes iteration count.

**Fig. 19:**
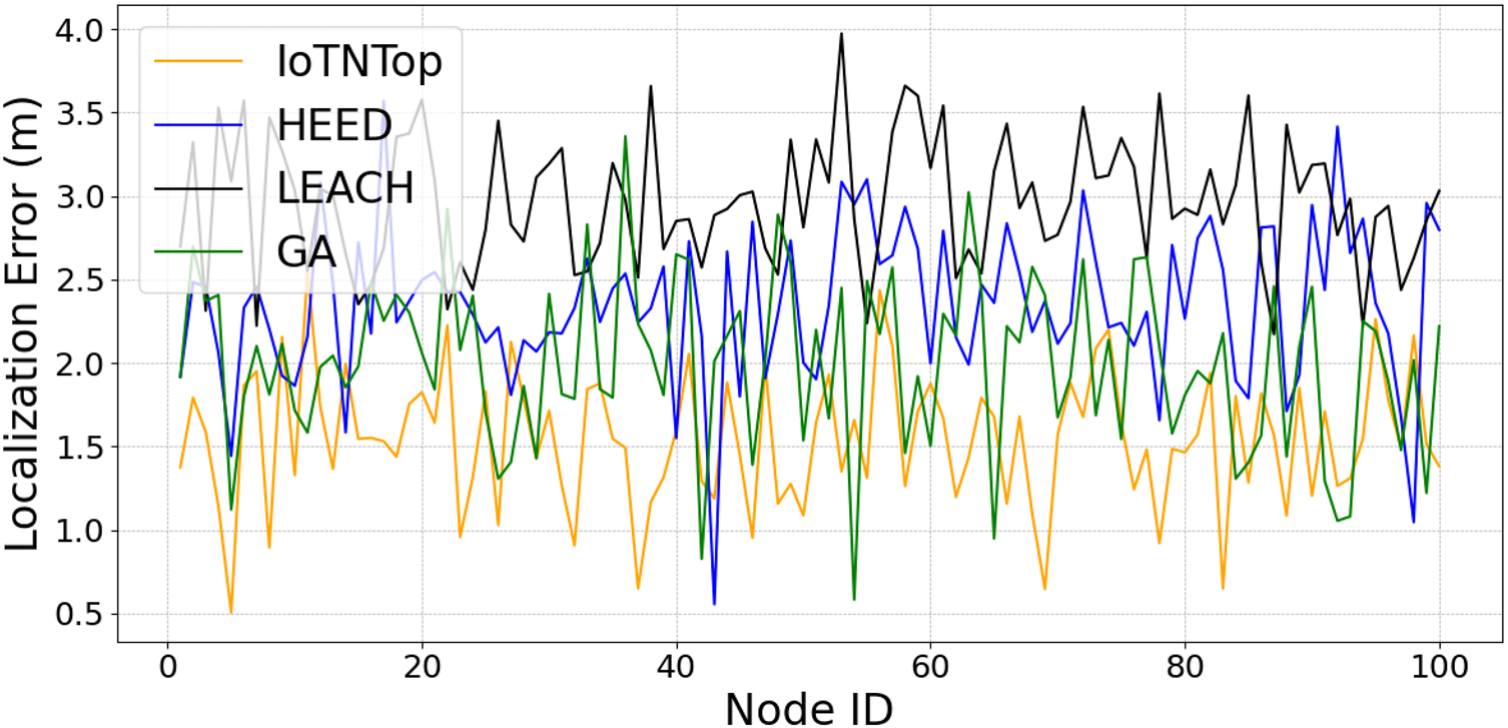
Localization error distribution across all nodes in the network after graph embedding and sub-graph alignment. Localization error is computed as the Euclidean distance between estimated and ground truth node positions.

The Node Performance Heatmap in Fig. 17 integrates three metrics–remaining energy, PDR, and error probability–providing a holistic visual of each node’s performance. Nodes that perform well across all dimensions appear in cool blue-green hues, while under-performing nodes (e.g., with high error or low energy) are flagged in warmer colors. The heatmap further enables rapid visual identification of bottlenecks, vulnerable nodes, or regions needing intervention (e.g., adding a gateway or increasing transmit power). The plot in Fig. 18 demonstrates that IoTNTop achieves higher average SNR more rapidly and consistently across iterations. This validates the numerical results in the paper (see Figs. 5 and 6), which show that IoTNTop converges in fewer iterations than competing methods. While LEACH and HEED were designed for simplicity and energy efficiency, they do not scale well in terms of error resilience or range optimization. These limitations become starkly visible when compared against IoTNTop’s multi-layered optimization approach.

**Fig. 20:**
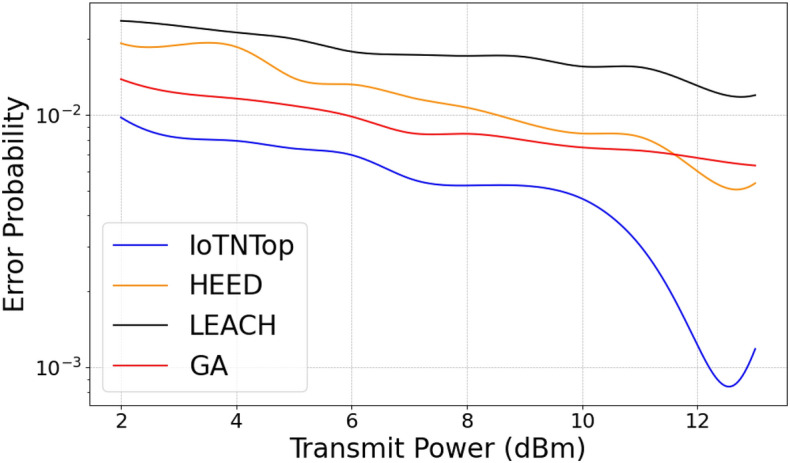
Variation in symbol error probability as a function of transmit power. The horizontal axis represents transmission power levels in dBm, and the vertical axis shows the corresponding symbol error probability.

**Fig. 21:**
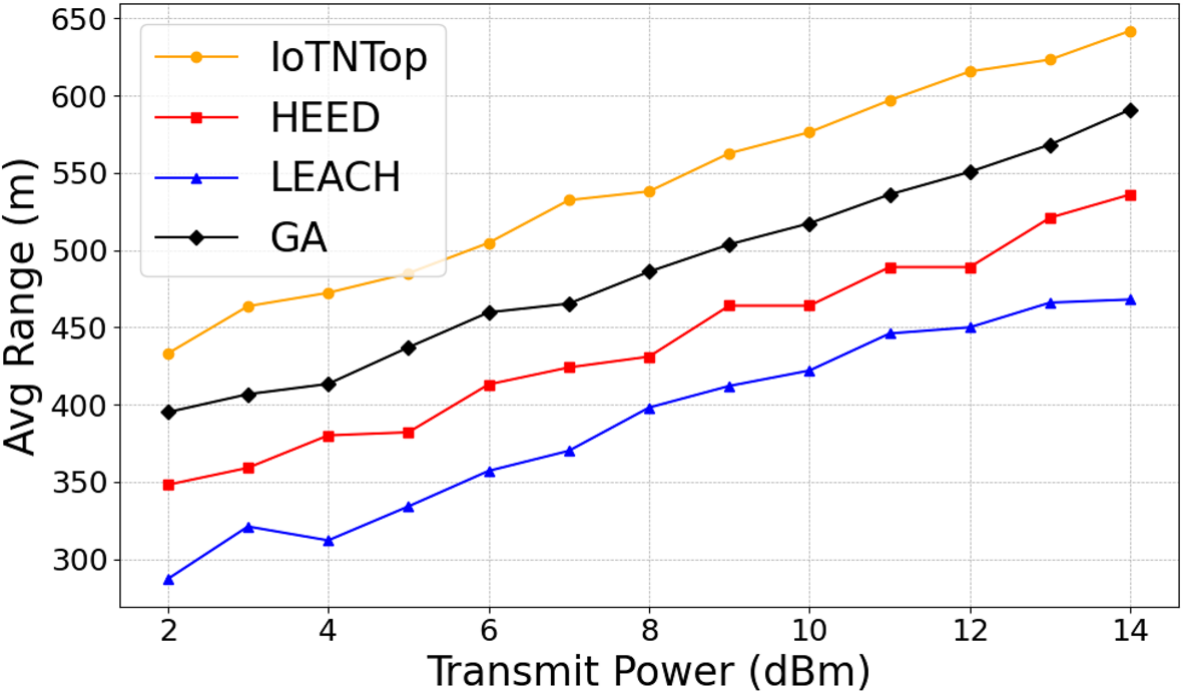
Achievable average transmission range per unit of transmit power across different topology strategies. The vertical axis represents range achievable in meters and the horizontal axis lists the algorithms evaluated.

In Fig. 19, IoTNTop consistently outperforms all other methods in maintaining low localization error across nodes. This supports the paper’s results showing improved accuracy in graph embedding and sub-graph stitching (as discussed in Sections II-B and II-C). LEACH and HEED, which use simpler heuristics for cluster formation, demonstrate higher errors due to their lack of spatial awareness, namely, they do not account for physical node positions, inter-node distances, or geometric consistency when forming clusters or assigning communication paths. Furthermore, they lack measurement refinement steps, such as eigenvector synchronization, reflection correction, or code-aware distance alignment, which are central to IoTNTop’s accurate graph realization. The relatively good performance of GA suggests its capability to adapt, but it still lacks the structural rigor, namely, explicit enforcement of geometric constraints, global consistency in sub-graph alignment, and direct incorporation of communication-centric metrics such as SNR, code rate, or error probability into its optimization objective. Unlike IoTNTop, which leverages graph realization principles and synchronized sub-graph stitching, GA operates as a general-purpose optimizer and may converge to topologies that are locally good but globally inconsistent or suboptimal in terms of error robustness and communication quality. The results in Fig. 20 show a significant drop in error probability as transmit power increases for all protocols, but IoTNTop consistently maintains the lowest error rates across all power levels. This aligns directly with the analytical results in the paper (Figs. 8, 9, 10), which illustrate IoTNTop’s ability to minimize transmission errors by jointly optimizing transmit power and code rate. The logarithmic scale highlights how IoTNTop achieves exponential improvements, reinforcing the effectiveness of its error-centric design.

Finally, Fig. 21 confirms that IoTNTop can achieve a longer communication range for a given transmit power compared to the other protocols. This supports the claims in the paper (Figs. 7, 8) that IoTNTop balances energy efficiency with network coverage. LEACH and HEED achieve shorter ranges, indicating suboptimal power utilization, while GA performs moderately but lacks the adaptive precision that IoTNTop achieves via its greedy edge selection and convergence-checking strategy. One key insight is that optimizing error probability and code rate jointly, as IoTNTop does, leads to superior results in all other areas (SNR, power efficiency, and range). Most legacy protocols treat these metrics independently, which appears suboptimal for different IoT application scenarios.

It is worth noting the “zigzag” behavior seen in Figs. 16,  18, and  19 and to a lesser degree in Figs. 20 and 21. These fluctuations are a direct result of the discrete nature of topology formation in the presence of dynamic network parameters. Small changes in input conditions can lead to abrupt shifts in the selected topology–such as inclusion/exclusion of certain links or nodes–thereby causing non-monotonic variations in metrics like code rate and error probability. This is further amplified by randomized node deployments, which may produce topology-sensitive outliers despite averaging across multiple simulation runs. Such behavior is expected in combinatorial optimization settings and reflects the sensitivity of real-world IoT deployments to small changes in physical or network-layer conditions.

## Conclusions

This paper tackles the challenge of designing IoT network topologies to maximize coverage and throughput with minimal number of devices while addressing issues like poor link quality and interference. By introducing a novel graph-realization approach that incorporates local distance information into the global network structure, we optimize the placement of end-nodes and gateways, improving information rates, and reducing transmit power and error probability. The methodology divides the network into sub-graphs, aligning them to form an efficient global structure. Through advanced mathematical modeling and algorithms, this scalable, noise-resilient solution outperforms traditional methods in minimizing errors and maximizing code rates, making it ideal for large, heterogeneous IoT networks. Across matched conditions used in our simulations, $N=100$ nodes over a $5\times 5\,\textrm{km}^{2}$ field with average transmit power of $5\,\textrm{dB}$ per node, *IoTNTop* achieves consistently better communication, localization tradeoffs than heuristic and exhaustive baselines. Most nodes maintain symbol error probability $<15\%$ (Fig. 15), and the network retains 60–$80\%$ of the initial per-node energy budget (Fig. 16) while sustaining higher achievable code rates across iterations and network sizes (Fig. 12). These trends persist for $N\in [50,300]$ (Figs. 12, 13, 14, 15, 16), and the algorithm typically converges in fewer iterations than baselines, reducing overall computational effort. Our present formulation assumes quasi-static deployments with partial but stationary distance/noise statistics; accuracy degrades when distance measurements are severely biased (like, persistent NLOS) or SNR estimates drift over time. We do not model mobility, clock drift, or rapid channel fading explicitly in this version; incorporating temporal dynamics, robust bias mitigation, and online recalibration are important directions. In future, we will explore : (i) adaptive re-embedding under time-varying channels and mobility, (ii) field trials with heterogeneous hardware to assess measurement bias and synchronization effects, and (iii) hybrid learning-based predictors that warm-start edge selection while preserving the error-centric guarantees.

## Supplementary Information

Supplementary Information.

## Data Availability

All data generated in this paper are simulation-based. The datasets generated and/or analyzed during the current study will be available from the corresponding author on reasonable request.
