# Supplementary material for: Optimized topology control for large-scale IoT networks using graph-based localization
Source: Sci Rep. 2026 Mar 17;16:13810. doi: 10.1038/s41598-026-43621-6 (PMC13129079; doi:10.1038/s41598-026-43621-6)
Supplement: Supplementary file 1 — Supplementary Information. [file 41598_2026_43621_MOESM1_ESM.pdf]

Appendices

A Optamility of (11)

a) *Objective Function Analysis*: The objective function is $\sum_{i=1}^{m} ||\bar{\mathsf{s}}e^{\imath\theta}(\bar{x}_i - \mu_{\bar{X}}) + (\bar{t} - \mu_{\bar{Y}}) - (\bar{y}_i - \mu_{\bar{Y}})||^2$. This function measures the squared Euclidean distance between the transformed $\bar{x}_i$ points and the $\bar{y}_i$ points.
b) *Define the Transformation*: Let s be the scaling factor, θ be the rotation angle, and $\bar{t}$ be the translation vector. The transformation applied to $\bar{x}_i$ is $\bar{\mathsf{s}}e^{\imath\theta}(\bar{x}_i - \mu_{\bar{X}}) + (\bar{t} - \mu_{\bar{Y}})$.
c) *Optimality Conditions*: To find the optimal s, θ, and $\bar{t}$, we need to minimize the sum of squared differences. We achieve this by finding the values that minimize the distance between the transformed points and the target points.
d) *Derivation of Optimal* $\bar{t}$: The optimal translation $\bar{t}$ aligns the centroids of the transformed $\bar{x}_i$ points and the $\bar{y}_i$ points. The centroids of the sets are $\mu_{\bar{X}}$ and $\mu_{\bar{Y}}$. Therefore, the optimal translation is $\bar{t} = \mu_{\bar{Y}} - \mathsf{s}e^{\imath\theta}\mu_{\bar{X}}$.
e) *Derivation of Optimal* s *and* θ: The optimal scaling and rotation can be derived using Singular Value Decomposition (SVD) of the centered data matrices. Let X_c and Y_c be the centered data matrices:

$$X_c = [\bar{x}_1 - \mu_{\bar{X}}, \bar{x}_2 - \mu_{\bar{X}}, \ldots, \bar{x}_m - \mu_{\bar{X}}] \tag{1}$$

$$Y_c = [\bar{y}_1 - \mu_{\bar{Y}}, \bar{y}_2 - \mu_{\bar{Y}}, \ldots, \bar{y}_m - \mu_{\bar{Y}}] \tag{2}$$

The cross-covariance matrix H is: $H = X_c Y_c^T$. Performing SVD on H: $H = U\Sigma V^T$. The optimal rotation matrix R is given by: $R = VU^T$. The optimal scaling factor s is $\mathsf{s} = \frac{\text{trace}(Y_c^T R X_c)}{\text{trace}(X_c^T X_c)}$. Final Optimal Values include,

- The optimal rotation angle θ is derived from the rotation matrix R: $e^{\imath\theta} = R$
- The optimal scaling factor is s: $\mathsf{s} = \frac{\text{trace}(Y_c^T R X_c)}{\text{trace}(X_c^T X_c)}$
- The optimal translation vector $\bar{t}$ is: $\bar{t} = \mu_{\bar{Y}} - \mathsf{s}e^{\imath\theta}\mu_{\bar{X}}$

By aligning the centroids, applying the optimal rotation, and scaling, we minimize the objective function. The optimal transformation parameters s, $e^{\imath\theta}$, and $\bar{t}$ achieve the minimum sum of squared differences, proving the optimality of the given problem.

B Optimality of (15)

Let $\mathbf{0}$ be a zero vector of appropriate dimension and e_i and e_j be the i-th and j-th standard basis vectors, respectively. Then:

$$(\mathbf{0}; e_i - e_j) = \begin{bmatrix} \mathbf{0} \\ e_i - e_j \end{bmatrix} \tag{3}$$

Consequently:

$$(\mathbf{0}; e_i - e_j)(\mathbf{0}; e_i - e_j)^T = \begin{bmatrix} \mathbf{0} & \mathbf{0} \\ \mathbf{0} & e_i - e_j \end{bmatrix} \begin{bmatrix} \mathbf{0} & \mathbf{0} \\ \mathbf{0} & e_i - e_j \end{bmatrix}^T \tag{4}$$

This simplifies to:

$$\begin{bmatrix} \mathbf{0} & \mathbf{0} \\ \mathbf{0} & (e_i - e_j)(e_i - e_j)^T \end{bmatrix} \tag{5}$$

The term $(e_i - e_j)(e_i - e_j)^T$ is a rank-1 matrix with non-zero elements only in the i-th and j-th positions. The constraint $(\mathbf{0}; e_i - e_j)(\mathbf{0}; e_i - e_j)^T\chi = d_{ij}^2$ implies that χ must be such that when pre-multiplied and post-multiplied by $(\mathbf{0}; e_i - e_j)$, the result is d_{ij}^2. This enforces a specific structure on χ. Specifically: $(\mathbf{0}; e_i - e_j)^T\chi(\mathbf{0}; e_i - e_j) = d_{ij}^2$. The matrix equation simplifies to $(e_i - e_j)^T\chi(e_i - e_j) = d_{ij}^2$. Since e_i and e_j are standard basis vectors, χ must have the property that $\chi_{ii} - 2\chi_{ij} + \chi_{jj} = d_{ij}^2$. This equation ensures that the squared distance d_{ij}^2 between the i-th and j-th positions is maintained. Given that the objective function is a constant zero, the problem reduces to finding χ such that the constraint $(e_i - e_j)^T\chi(e_i - e_j) = d_{ij}^2$ is satisfied. This constraint enforces that the matrix χ correctly represents the distances d_{ij} between points i and j. The constraints are linear in nature and define a feasible region. Since the objective function is zero and does not change, any χ that satisfies the constraint is optimal. Therefore, the problem is optimal because the constraints alone define the feasible set, and any χ that satisfies the constraint will achieve the objective function value of zero, proving the optimality.

C Optimality of (30)

To find the optimal $\mathbb{Q}_{ji}$ and h_{ja}, let's solve the optimization problem step-by-step: The optimization problem is:

$$\begin{aligned} \text{minimize}_{\mathbb{Q}_{ji}} \quad & \mathcal{G}(\Phi) = \left[\frac{\mathsf{r}_j^{\nu}}{\mathcal{E}_j} + d_{ji}^{\nu}\right] \times \mathbb{Q}_{ji} \\ \text{subject to,} \quad & \mathbb{Q}_{ji} = \max_{a\in V}(\mathsf{h}_{ja}) \\ & 0 \leq \mathsf{h}_{ja} \leq \mathcal{R}_{ja}, 0 < \mathsf{r}_j \leq \mathsf{r}_{\max} \\ & \sum_{a|a\in V} \mathsf{h}_{ja} - \sum_{a|j\in V} \mathsf{h}_{aj} = \psi_j \text{ for } d_{ji} \leq \mathsf{r}_j \end{aligned} \tag{6}$$

The Lagrangian is given by:

$$\begin{aligned} \mathcal{L} = & \left[\frac{\mathsf{r}_j^{\nu}}{\mathcal{E}_j} + d_{ji}^{\nu}\right] \times \mathbb{Q}_{ji} + \lambda_1(\mathbb{Q}_{ji} - \max_{a\in V}(\mathsf{h}_{ja})) \\ & + \sum_{a\in V} \lambda_{2a}(\mathsf{h}_{ja} - \mathcal{R}_{ja}) + \sum_{a\in V} \lambda_{3a}(-\mathsf{h}_{ja}) + \lambda_4(\mathsf{r}_j - \mathsf{r}_{\max}) \\ & + \lambda_5(-\mathsf{r}_j) + \lambda_6\left(\sum_{a\in V} \mathsf{h}_{ja} - \sum_{a\in V} \mathsf{h}_{aj} - \psi_j\right) \end{aligned} \tag{7}$$

Using the Karush-Kuhn-Tucker (KKT) conditions we formulate;
a) *Stationarity*:

$$\frac{\partial \mathcal{L}}{\partial \mathbb{Q}_{ji}} = \left[\frac{\mathsf{r}_j^{\nu}}{\mathcal{E}_j} + d_{ji}^{\nu}\right] + \lambda_1 = 0 \quad \Rightarrow \quad \lambda_1 = -\left[\frac{\mathsf{r}_j^{\nu}}{\mathcal{E}_j} + d_{ji}^{\nu}\right] \tag{8}$$

$$\frac{\partial \mathcal{L}}{\partial \mathsf{h}_{ja}} = -\lambda_1 \frac{\partial(\max_{a\in V}(\mathsf{h}_{ja}))}{\partial \mathsf{h}_{ja}} + \lambda_{2a} - \lambda_{3a} + \lambda_6 = 0 \tag{9}$$

Note: The partial derivative $\frac{\partial(\max_{a\in V}(\mathsf{h}_{ja}))}{\partial \mathsf{h}_{ja}}$ is 1 if $\mathsf{h}_{ja} = \max_{a\in V}(\mathsf{h}_{ja})$ and 0 otherwise.

$$\begin{aligned} \frac{\partial \mathcal{L}}{\partial \mathsf{r}_j} &= \frac{\nu \mathsf{r}_j^{\nu-1}}{\mathcal{E}_j}\mathbb{Q}_{ji} - \lambda_4 + \lambda_5 = 0 \\ \Rightarrow \quad \lambda_4 - \lambda_5 &= \frac{\nu \mathsf{r}_j^{\nu-1}}{\mathcal{E}_j}\mathbb{Q}_{ji} \end{aligned} \tag{10}$$

b) *Primal Feasibility*:
The original constraints should hold:

- $\mathbb{Q}_{ji} = \max_{a\in V}(\mathsf{h}_{ja})$
- $0 \leq \mathsf{h}_{ja} \leq \mathcal{R}_{ja}$
- $0 < \mathsf{r}_j \leq \mathsf{r}_{\max}$
- $\sum_{a\in V} \mathsf{h}_{ja} - \sum_{a\in V} \mathsf{h}_{aj} = \psi_j$ for $d_{ji} \leq \mathsf{r}_j$

c) *Dual Feasibility*: $\lambda_i \geq 0$ for all i.
d) *Complementary Slackness*: Each λ_i should be zero if the corresponding constraint is strictly less than its bound.
e) *Solving for Optimal Values*:

- From the stationarity condition with respect to $\mathbb{Q}_{ji}$:

$$\lambda_1 = -\left[\frac{\mathsf{r}_j^{\nu}}{\mathcal{E}_j} + d_{ji}^{\nu}\right] \tag{11}$$

- From the stationarity condition with respect to h_{ja}:
  If $\mathsf{h}_{ja} = \max_{a\in V}(\mathsf{h}_{ja})$: $-\lambda_1 + \lambda_{2a} - \lambda_{3a} + \lambda_6 = 0$
  Otherwise: $\lambda_{2a} - \lambda_{3a} + \lambda_6 = 0$.

- From the stationarity condition with respect to r_j: $\lambda_4 - \lambda_5 = \frac{\nu \mathsf{r}_j^{\nu-1}}{\mathcal{E}_j}\mathbb{Q}_{ji}$. Given that $\mathbb{Q}_{ji} = \max_{a\in V}(\mathsf{h}_{ja})$, we can determine that $\mathbb{Q}_{ji}$ is maximized when h_{ja} is at its upper bound $\mathcal{R}_{ja}$: $\mathbb{Q}_{ji} = \mathcal{R}_{ja} \quad \text{where} \quad \mathsf{h}_{ja} = \max_{a\in V}(\mathsf{h}_{ja})$.

f) *Summary of Optimal Values*:

- $\mathbb{Q}_{ji} = \mathcal{R}_{ja}$
- $\mathsf{h}_{ja} = \mathcal{R}_{ja}$ if $\mathsf{h}_{ja} = \max_{a\in V}(\mathsf{h}_{ja})$

Thus, the optimal solution to the given problem is to set $\mathbb{Q}_{ji}$ equal to the maximum allowable rate $\mathcal{R}_{ja}$ for the flow[1] h_{ja} and ensure all constraints are satisfied.

[1]denotes the number of encoded information symbols sent per unit time over the link from node j to node i reflecting the data load or routing weight assigned to that link
